# PP2Ac Regulates Autophagy via Mediating mTORC1 and ULK1 During Osteoclastogenesis in the Subchondral Bone of Osteoarthritis

**DOI:** 10.1002/advs.202404080

**Published:** 2024-07-23

**Authors:** Haifeng Zhang, Gaoran Ge, Wei Zhang, Houyi Sun, Xiaolong Liang, Yu Xia, Jiacheng Du, Zerui Wu, Jiaxiang Bai, Huilin Yang, Xing Yang, Jun Zhou, Yaozeng Xu, Dechun Geng

**Affiliations:** ^1^ Department of Orthopedics Surgery the First Affiliated Hospital of Soochow University Suzhou Jiangsu 215006 China; ^2^ Department of Orthopaedic Surgery Shanghai General Hospital Shanghai Jiao Tong University School of Medicine Shanghai 200080 China; ^3^ Department of Orthopedics Qilu Hospital of Shandong University Jinan Shandong 250063 China; ^4^ Department of Biochemistry and Molecular Biology Jeonbuk National University Medical School Jeonju Jeonbuk 54896 South Korea; ^5^ Department of Orthopedics Changshu Hospital Affiliated to Soochow University Changshu Jiangsu 215501 China; ^6^ Department of Orthopedics the First Affiliated Hospital of USTC Division of Life Sciences and Medicine University of Science and Technology of China Hefei Anhui 234000 China; ^7^ Orthopedics and Sports Medicine Center Suzhou Municipal Hospital Nanjing Medical University Affiliated Suzhou Hospital 242, Guangji Road Suzhou Jiangsu 215008 China

**Keywords:** autophagy, dephosphorylation, mTORC1, osteoclastogenesis, PP2Ac, ULK1

## Abstract

The molecular mechanism underlying abnormal osteoclastogenesis triggering subchondral bone remodeling in osteoarthritis (OA) is still unclear. Here, single‐cell and bulk transcriptomics sequencing analyses are performed on GEO datasets to identify key molecules and validate them using knee joint tissues from OA patients and rat OA models. It is found that the catalytic subunit of protein phosphatase 2A (PP2Ac) is highly expressed during osteoclastogenesis in the early stage of OA and is correlated with autophagy. Knockdown or inhibition of PP2Ac weakened autophagy during osteoclastogenesis. Furthermore, the ULK1 expression of the downstream genes is significantly increased when PP2Ac is knocked down. PP2Ac‐mediated autophagy is dependent on ULK1 phosphorylation activity during osteoclastogenesis, which is associated with enhanced dephosphorylation of ULK1 Ser637 residue regulating at the post‐translational level. Additionally, mTORC1 inhibition facilitated the expression level of PP2Ac during osteoclastogenesis. In animal OA models, decreasing the expression of PP2Ac ameliorated early OA progression. The findings suggest that PP2Ac is also a promising therapeutic target in early OA.

## Graphical abstract

The schematic illustrates how PP2Ac mediates autophagy during osteoclastogenesis in the subchondral bone in early osteoarthritis (OA). PP2Ac plays a significant role in regulating autophagy through its involvement in mTORC1 and the phosphorylation of ULK1. Attenuating PP2Ac expression during osteoclastogenesis can potentially delay the progression of OA via inhibiting autophagy.

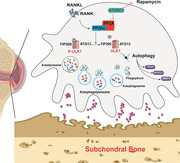

## 1 Introduction

Osteoarthritis (OA) is a prevalent degenerative whole‐joint disease that involves abnormalities in articular cartilage, subchondral bone remodeling, synovial inflammation, and osteophyte formation.^[^ ^1^ ^]^ Among these pathological lesions, the disruption of subchondral bone homeostasis and the imbalance of bone metabolism caused by abnormal osteoclastogenesis contributed to the pathogenesis of OA, particularly in the early stage.^[^ ^2^ ^]^ However, the exact mechanism remains unclear.

Autophagy (macroautophagy), as a conserved self‐digestion process, could maintain cellular homeostasis and viability under environmental stress.^[^ ^3^ ^]^ Previous studies have pointed receptor activator of nuclear factor kappa‐B ligand (RANKL) mediates osteoclastogenesis, and autophagy has a positive effect on promoting osteoclast activity when RANKL binds to the osteoclast progenitors RANK receptor.^[^ ^4^ ^]^ During osteoclastogenesis, RANKL by activating the autophagolysosomal degradation of TRAF3 in osteoclast precursors (OCPs) promote them to differentiate into mature osteoclasts, leading to the initiation of bone remodeling.^[^ ^5^ ^]^ Autophagy has also been involved in regulating osteoclast function and bone metabolism, including osteoclast migration, differentiation, and bone resorption,^[^ ^6^ ^]^ and our previous research showed increased numbers of osteoclasts and enhanced bone‐resorbing activity in subchondral bone in a mouse model of OA.^[^ ^7^ ^]^ Therefore, further study is essential to understand how autophagy is involved in abnormal osteoclastogenesis and the underlying mechanisms initiated in early OA disease.

As the major serine/threonine protein phosphatase in eukaryotic cells, protein phosphatase 2A (PP2A) is involved in the regulation of an extensive network of cellular processes.^[^ ^8^ ^]^ PP2A encompasses multiple complexes constructed through the combination of regulatory family and scaffolding subunits with a common catalytic subunit (PP2Ac), which mainly participates in regulating post‐translational phosphorylation levels.^[^ ^9^ ^]^ Dysregulation of PP2A signaling pathways contributes to various diseases, such as diabetes, neurodegenerative diseases, and cancer.^[^ ^10^ ^]^ One major pathogenic mechanism is the PP2A‐mediated regulation of autophagy, which is complex and multifaceted and depends on the multi‐component structure of PP2A. Among them, the PP2A catalytic subunit consisting of α (PPP2CA) and β (PPP2CB) isoforms contains a phosphatase domain and plays an important regulatory role in the holoenzyme.^[^ ^11^ ^]^ Our preceding study revealed that PP2A was highly expressed in osteoclast‐mediated peri‐prosthetic osteolytic disease, and its pathogenesis was associated with abnormal osteoclastogenesis and enhanced bone‐resorbing activity.^[^ ^12^ ^]^ Interestingly, other studies suggested expression of PPP2CA in osteoblasts mediates osteoclast differentiation and osteoclastogenesis by regulating RANKL and osteoprotegerin (OPG),^[^ ^13^ ^]^ although the specific mechanism has not been elucidated. Evidence‐based on a link between PP2Ac and osteoclastogenesis, we hypothesized that PP2Ac could regulate osteoclastogenesis through autophagy in OA disease.

In this study, we first conducted single‐cell RNA sequence (scRNA‐seq) analysis based on human OA subchondral bone tissue and performed bulk RNA sequence analysis during osteoclastogenesis to identify differentially expressed genes (DEGs), and then validated the high expression of PP2Ac in human knee joint tissue, rat OA specimens, and in the process of osteoclastogenesis. The alterations at autophagy levels during osteoclastogenesis were detected using knocking down or inhibiting PP2Ac with BMMs and RAW264.7. After knocking down PP2Ac in BMMs, the downstream target molecules were examined by gene transcriptome sequencing, and verified the interaction between PP2Ac and ULK1. We also found that during osteoclastogenesis, mechanistic target of rapamycin complex 1 (mTORC1) regulates the phosphorylation balance of ULK1 by affecting the PP2Ac activity. Finally, pharmacological inhibition of PP2Ac was performed in the early stages of OA to assess its therapeutic effect. Our study might provide important insights into the autophagy‐related molecular mechanisms underlying osteoclastogenesis leading to OA and identify PP2Ac as a potential target for designing therapies.

## 2 Results

### 2.1 Up‐Regulation of PP2Ac Expression in Osteoclasts of Subchondral Bone in OA

**Figure 1:**
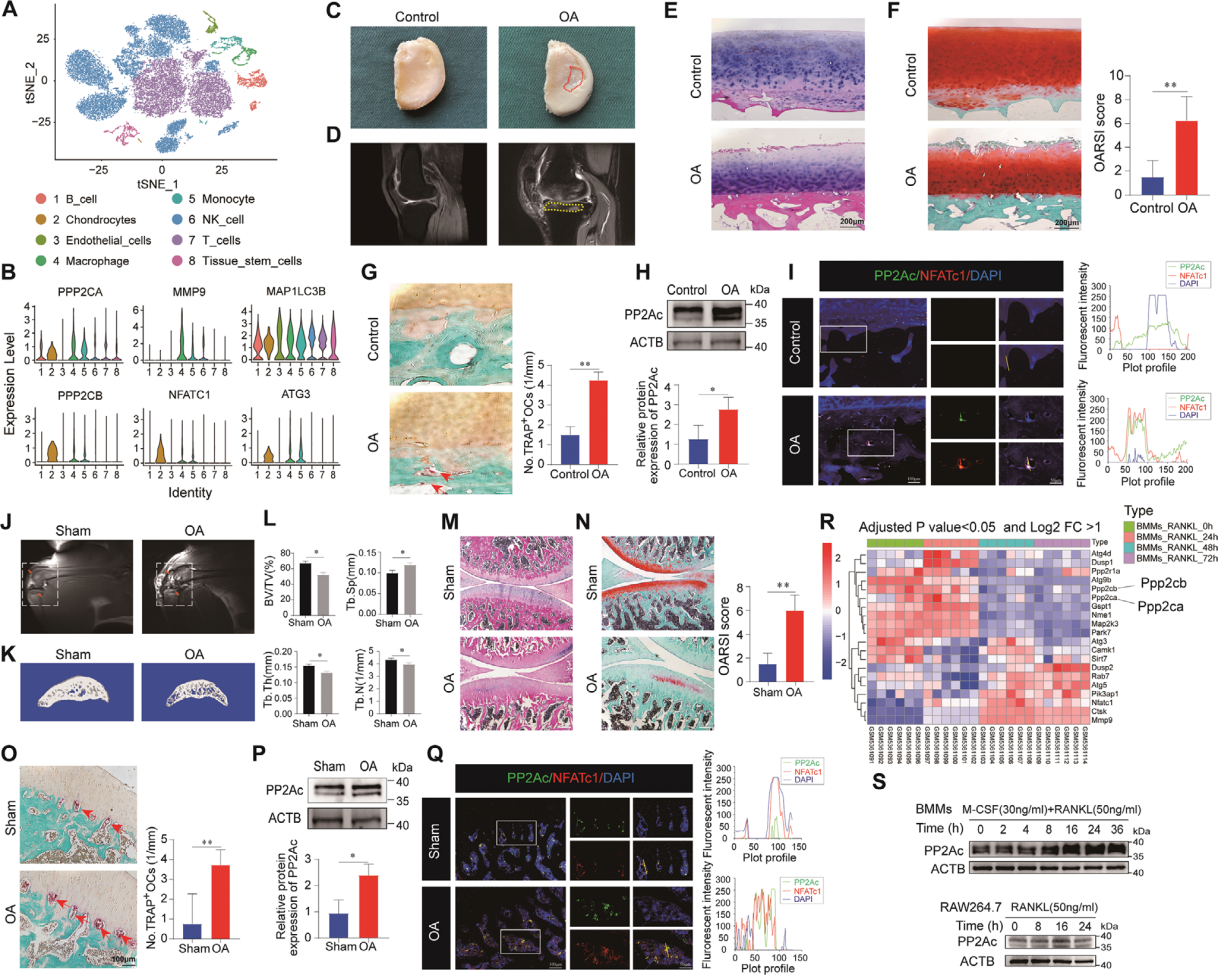
Increased PP2Ac expression of osteoclast in subchondral bone with OA disease. A) A t‐distributed stochastic neighbor embedding (t‐SNE) plot to visualize the eight main sub‐clusters of cells identified in the subchondral bone with OA. B) Violin plots showing normalized expression levels of marker genes across different cell subpopulations. C) The gross appearance of human knee joint specimens in the medial tibial plateau between the control and OA groups. The red‐lined areas represent severe wear in the OA group. D) T2‐weighted magnetic resonance imaging (MRI) detection of articular cartilage and subchondral bone lesions in two groups. E) H&E staining revealed the histology changes between the two groups. F) Safranin O fast green staining and analysis of the tibial Osteoarthritis Research Society International (OARSI) scores to indicate the level of cartilage destruction and severity of OA. G) Tartrate‐resistant acid phosphatase (TRAP) assay showing the activity of osteoclasts at the tibial subchondral bone in both the control and OA groups. H) Western blot assay revealing the gene expression of PP2Ac originated from the human specimen. I) Immunofluorescence co‐staining and analysis of fluorescence intensity to examine the expression of PP2Ac and NFATc1 in the subchondral bone of the human knee joint. J) T2‐weighted MRI to detect changes in the rat knee joint following ACLT surgery at 2‐week time points in both sham and OA groups. K) Micro‐CT 3D reconstruction image of tibial subchondral bone medial compartment for both sham and OA groups. L) Quantitative analysis of bone volume fraction (BV/TV), trabecular number (Tb.N), trabecular thickness (Tb.Th), and trabecular separation (Tb.Sp) for both groups. M) H&E staining revealing the gross histological alteration in rat OA model. N) Safranin O fast green staining and OARSI score exhibiting and assessing the damage level in articular cartilage. O) TRAP staining observation and analysis in the tibial subchondral bone between the control and OA model pointing to the number and activity of osteoclasts. P) Western blot assay showing the PP2Ac expression levels in subchondral bone tissue from the knee joints of sham and rats OA groups. Q) Immunofluorescence co‐localization and fluorescence intensity analysis of PP2Ac and NFATc1 expression in subchondral bone within two groups of rat model. R) Heatmap visualized the expression of differentially expressed genes (DEGs) during osteoclastogenesis by comparing gene expression levels in bone marrow‐derived macrophages (BMMs) at 0, 24, 48, and 72 h after RANKL induction. S) Western blot assay was performed to detect PP2Ac expression in OCPs (BMMs and Raw264.7) following RANKL induction at different time points during osteoclastogenesis. (*n* = 3, mean ± SD; *p < 0.05; **p < 0.01; vs control group or sham group).

To identify PP2Ac expression, a powerful scRNA‐seq analysis on human OA subchondral bone tissue was performed. After filtering and normalizing the GSE196678 dataset, 19 clusters were generated (Figure S1A, Supporting Information), and eight‐cell types were identified after cell subset annotation, including B cells, chondrocytes, endothelial cells, macrophages, monocytes, NK cells, T cells, and tissue stem cells (**Figure** **1**A). Among these, osteoclasts are derived from macrophage/monocyte precursor cells, and then the macrophage cell subgroup was selected for the following analysis. Comparing HVGs between subgroups, violin plots indicated high expression of osteoclast markers (MMP9 and NFATC1), autophagy‐related markers (MAP1LC3 and ATG3), and PP2Ac including PPP2CA and PPP2CB in the macrophage cell subtype (Figure 1B). And feature map showed the distribution of the above marker genes (Figure S1B, Supporting Information), and dot plots exhibited more osteoclasts and autophagy‐related genes (Figure S1C, Supporting Information). To further verify PP2Ac expression in the human knee joint, we selected the medial tibial plateau from the young amputee patients (control group) and early OA specimens derived from medial unicondylar knee arthroplasty (OA group, Figure 1C). We found higher signal lesions in the subchondral bone region on MRI T2‐weighted images in the OA group compared to the control group (Figure 1D). H&E staining revealed roughness and cracking of the cartilage surface, accompanied by aberrant subchondral bone change in OA samples (Figure 1E). Safranin O fast green staining indicated a loss of glycosaminoglycans and aggrecan in articular cartilage, with higher OARSI scores observed in the OA group (Figure 1F). The number of TRAP‐positive osteoclasts in the subchondral bone was increased in the OA group, while fewer positive‐stained osteoclasts were observed in the control group (Figure 1G). The western blot assay revealed up‐regulation of PP2Ac expression in the OA group at subchondral bone sites (Figure 1H). The PP2Ac co‐staining with NFATc1 suggested that PP2Ac was functionally upregulated in early OA. Fluorescence intensity curves confirmed that PP2Ac and NFATc1 overlapped with higher expression levels in the OA group but not in the control group (Figure 1I).

To further confirm the PP2Ac expression in animal models, anterior cruciate ligament transection (ACLT) surgery was performed to induce an OA model in SD rats at different time points. H&E stain (Figure S1D, Supporting Information) revealed progressively rougher articular cartilage surfaces and increasingly irregular subchondral bone from the first to eighth week. Safranin O fast green staining (Figure S1E,G, Supporting Information) indicated a progressively increasing loss of proteoglycans and a gradually rising OARSI score. Interestingly, the number of TRAP‐positive osteoclasts increased significantly in the second week of OA according to cell counting (Figure S1F,H, Supporting Information). Micro‐CT reconstruction also showed relatively sparse subchondral bone trabeculae in the second week, and after quantitatively evaluating bone tissue parameters, BV/TV, Tb.Th and Tb.N also decreased at two weeks post‐operation, while Tb.Sp increased at this time point (Figure S1I, Supporting Information). These results indicated the feasibility of the animal OA model and showed that osteoclasts were most active at two weeks of OA, and gradually decreased over time. Then MRI and micro‐CT scans showed abnormally high signals in the cartilage layer microstructural damage to the subchondral bone and decreased bone density in the OA group, respectively (Figure 1J–L). Histological staining, including H&E, Safranin O fast green, and TRAP staining, showed that the cartilage layer was damaged and destroyed, and the osteoclastic activity of subchondral bone was increased in the OA group of rats (Figure 1M–O). We further found above similar experimental outcomes to human OA disease in the rat sham group and the 2‐week OA group. Additionally, we conducted a western blot assay (Figure 1P) and immunofluorescence co‐staining of PP2Ac with NFATc1 (Figure 1Q), which revealed the higher expression of PP2Ac in osteoclasts in subchondral bone also existed in the rat OA model.

At the cellular level, the DEGs were screened in RANKL‐induced BMMs for 0, 24, 48, and 72 h via the GSE176265 dataset. As depicted in Figure 1R, the expression of PP2Ac increased in the early 24 h after RANKL induction and decreased after this time point. The expression of PP2Ac during osteoclastogenesis was further established by inducing BMMs and RAW264.7 cells with M‐CSF and/or RANKL at different time points. Through immunofluorescence experiments, we found that the highest expression of PP2Ac occurred at 16 h during osteoclastogenesis (Figure S1J, Supporting Information), consistent with western blot experiments (Figure 1S). Therefore, these findings revealed the association between high expression of PP2Ac and osteoclastogenesis in early OA disease.

### 2.2 Inhibition of PP2Ac Suppresses Osteoclastogenesis in OCPs

To investigate the impact of PP2Ac on osteoclastogenesis, we utilized siRNA PP2Ac or the pharmacological inhibitor Microcystin‐LR (MC‐LR) to suppress gene expression in BMMs and RAW264.7 cells. First, the effectiveness after PP2Ac knockdown was verified using western blot assay and GFP staining (Figure S2A–D, Supporting Information). The CCK8 assay screens out the safety and efficacy of the reagent concentration in BMMs (Figure S2E, Supporting Information).

**Figure 2:**
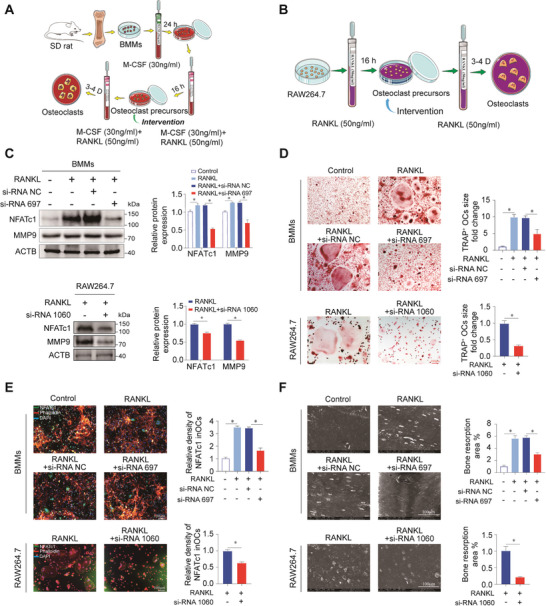
Inhibitory effect of PP2Ac inhibition on osteoclastogenesis. A) The flowchart illustrates the process of osteoclastogenesis using BMMs with M‐CSF and RANKL induction, followed by intervention with either PP2Ac inhibition or PP2Ac siRNA. B) The flowchart illustrates the process of osteoclastogenesis in RAW264.7 cell lines induced with RANKL and then intervened with PP2Ac siRNA. C) Western blot experiment and quantitative analysis to measure the expression of osteoclastic markers during osteoclastogenesis in BMMs and Raw264.7 cells with PP2Ac inhibition. D) The size of TRAP‐stained multinucleated cells was assessed in BMMs and Raw264.7 cells after 5 days of RANKL induction. E) Immunofluorescence staining of NFATc1 with phalloidin or F‐actin to examine the expression of osteoclast functional proteins in BMMs after 5 days of RANKL induction, followed by quantification. F) Bone plate resorption assay and quantification of the resorption area to evaluate the resorption function of osteoclast cells. (*n* = 3, mean ± SD; *p < 0.05; **p < 0.01; vs control group).

In flow chart **Figure** **2**A,B, BMMs, and RAW264.7 were induced for 16 h with RANKL, followed by intervention with PP2Ac siRNA or MC‐LR. Western blot assay outcomes showed that reducing PP2Ac expression also decreased the levels of osteoclast markers NFATc1 and MMP9 (Figure 2C; Figure S3A, Supporting Information). Furthermore, TRAP staining revealed that inhibiting osteoclastogenesis, decreasing osteoclast size, and reducing osteoclast area occurred with PP2Ac siRNA and MC‐LR intervention when OCPs were induced (Figure 2D; Figure S3B, Supporting Information). Co‐immunofluorescence staining of NFATc1 and F‐actin in osteoclasts indicated that the fluorescence intensity of NFATc1 decreased after PP2Ac knockdown or inhibition (Figure 2E; Figure S3C, Supporting Information). The bone plate resorption tests confirmed that knocking down or inhibiting PP2Ac expression attenuates osteoclast bone resorption capacity by measurement of bone resorption area (Figure 2F; Figure S3D, Supporting Information). Based on these results, it appears that PP2Ac can be involved in the regulation of osteoclastogenesis.

### 2.3 PP2Ac Regulated Autophagy During Osteoclastogenesis

**Figure 3:**
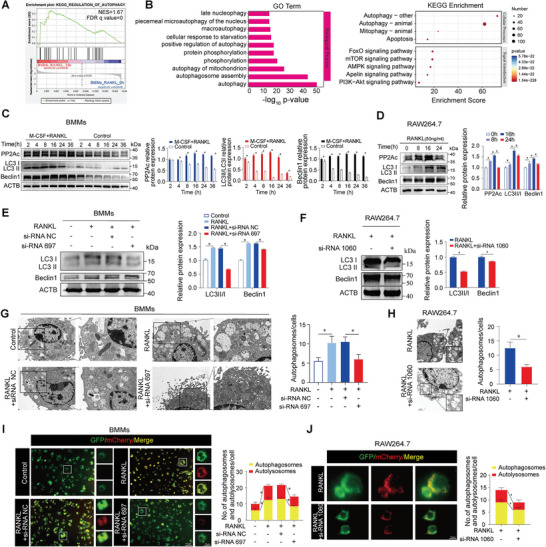
PP2Ac regulated autophagy during osteoclastogenesis. A) The GSEA software analysis showed enrichment of genes involved in the regulation of the autophagy pathway between the 0 and 24 h time points during osteoclastogenesis via the GSE176265. B) GO and KEGG enrichment pathway plots with the DEGs between 0 and 24 h. C, D) Western blot assay and quantitative analysis of gray value to detect the expression levels of PP2Ac and autophagy marker proteins at different time points following RANKL induction during osteoclastogenesis. E, F) Western blot analysis and quantitative assessment of autophagy marker expression in response to PP2Ac knockdown. G, H) Detection and comparison of autophagic vacuoles or autophagolysosomes in BMMs and RAW264.7 induced with RANKL using TEM after PP2Ac knockdown. I, J) The mCherry‐GFP‐LC3 staining and fluorescence intensity analysis to monitor autophagic flux in BMMs and RAW264.7 cells with PP2Ac knockdown. Autophagosomes were visualized with yellow dots (mCherry and GFP co‐localization) and autolysosomes were identified as red dots (mCherry). (*n* = 3, mean ± SD; *p < 0.05; **p < 0.01; vs control group).

To elucidate the correlation between PP2Ac and autophagy during osteoclastogenesis, GSEA enrichment analysis was performed on the GSE176265 database, suggesting that autophagy pathways were enriched after RANKL induction between 0 and 24 h (**Figure** **3**A). GO function and KEGG pathway analysis revealed that DEGs could enrich the autophagy, macroautophagy, mTOR, AMPK, and PI3K‐AKT signaling pathways (Figure 3B). According to these clues, BMMs and RAW264.7 were selected to induce with RANKL at different time points. We found that with increasing expression levels of PP2Ac, the expression trends of autophagy molecules LC3II/I and Beclin1 also altered (Figure 3C,D). This hinted the PP2Ac expression is closely related to autophagy. Western blot experiments also showed that the autophagy level of OCPs (BMMs and RAW264.7) was also reduced when PP2Ac expression was decreased (Figure 3E,F; Figure S4A, Supporting Information). TEM detection showed the number of autophagic vacuoles or autolysosomes decreased in the PP2Ac siRNA or MC‐LR groups compared with the RANKL‐induced group (Figure 3G,H; Figure S4B, Supporting Information). Additionally, mCherry‐GFP‐LC3 staining showed the number of autophagosomes and autolysosomes decreased after PP2Ac inhibition (Figure 3I,J; Figure S4C, Supporting Information). These results suggest that PP2Ac is involved in regulating osteoclastogenesis by mediating the autophagy pathway.

### 2.4 The Effects of Autophagy Regulation on Osteoclastogenesis

To explore the role of autophagy in RANKL‐induced osteoclastogenesis, pharmaceutical agents were used to block and enhance autophagy levels. The autophagy inhibitor (3‐Methyladenine, 3‐MA) and the activator (Rapamycin, RAPA) interfered with BMMs during osteoclastogenesis. Western blot experiments showed that 3‐MA intervention decreased the protein expression levels of LC3II/I and Beclin1 (Figure S5A, Supporting Information). TEM analysis showed that 3‐MA reduced autophagic vesicles or autolysosomes compared to the RANKL‐induced group (Figure S5B, Supporting Information). Furthermore, the mCherry‐GFP‐LC3 staining showed that 3‐MA reduced the number of autophagosomes and autophagolysosomes (Figure S5C, Supporting Information). In terms of the morphology and function of osteoclasts, the western blot assay indicated that 3‐MA reduced the expression levels of NFATc1 and MMP9 (Figure S5D, Supporting Information). TRAP staining also indicated that 3‐MA decreased the size and area of osteoclasts induced by RANKL (Figure S5E, Supporting Information). Immunofluorescence staining of osteoclasts revealed that 3‐MA inhibited the fluorescent expression of NFATc1 (Figure S5F, Supporting Information). Bone plate resorption tests confirmed that 3‐MA reduced the bone resorption activity of osteoclasts (Figure S5G, Supporting Information). On the other hand, as shown in Figure S5H–N (Supporting Information), RAPA promoted autophagy levels in BMMs during osteoclastogenesis, suggesting that autophagy is essential for activating osteoclastogenesis.

### 2.5 PP2Ac Regulates Autophagy through ULK1 Activation

**Figure 4:**
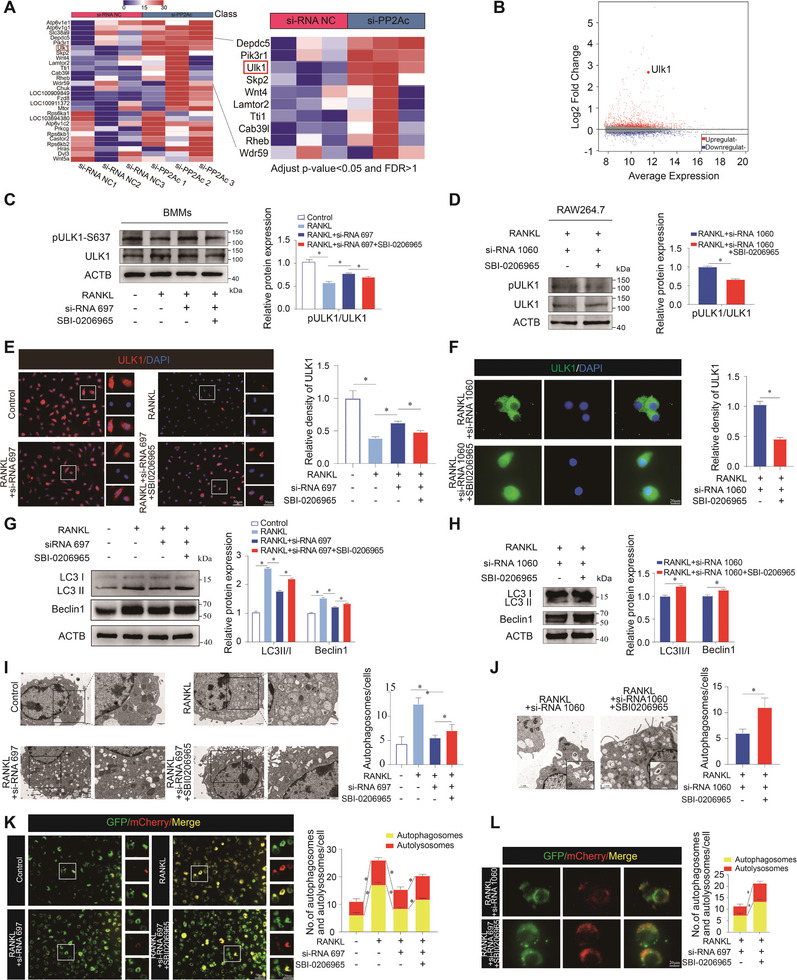
PP2Ac‐mediated regulation of autophagy is dependent on ULK1 activity. A) The heat map illustrates changes in gene expression levels of downstream DEGs following PP2Ac knockdown in RANKL‐induced BMMs, revealing a significant increase in ULK1 expression. B) The volcano plots depicted DEGs changes resulting from the intervention of PP2Ac siRNA, including the expression level of the ULK1 gene. C, D) The level of phosphorylated ULK1 protein expression after PP2Ac knockdown and ULK1 inhibition was analyzed by western blot assay and gray value analysis in BMMs and RAW264.7. E, F) Immunofluorescence staining and quantitative analysis of ULK1 expression level with PP2Ac knockdown and ULK1 inhibition. G, H) Western bolt assay and quantitative analysis to assess the expression levels of autophagy marker proteins in BMMs and RAW264.7 cells after PP2A knockdown and ULK1 inhibition. I, J) Transmission electron microscopy (TEM) and quantitative analysis were used to detect autophagosomes or autolysosomes after the addition of ULK1 inhibitor and knockdown of PP2A. K, L) The mCherry‐GFP‐LC3 staining and quantitative analysis about autophagosomes or autolysosomes after PP2Ac knockdown and ULK1 inhibition. (*n* = 3, mean ± SD; *p < 0.05; **p < 0.01; vs control group).

To elucidate the molecular mechanism of PP2Ac‐mediated autophagy during osteoclastogenesis, gene transcriptome sequencing was performed on PP2Ac siRNA knockdown with RANKL‐induced BMMs. Western blot assay revealed the reduced expression of PP2Ac after si‐PP2Ac knockdown under RANKL intervention (Figure S6A, Supporting Information). Heat map (**Figure** **4**A) and volcano plots (Figure 4B) showed an increase in ULK1 expression levels after PP2Ac knockdown. As a phosphatase, PP2Ac may be involved in regulating ULK1 phosphorylation. Previous studies have shown that PP2A may function at multiple phosphorylated residue sites of ULK1, such as Serine 17, Serine 556, Serine 637, Serine 757, and Serine 717.^[^ ^14^ ^]^ Through western blot experiments, our screening found that the phosphorylation level of Serine 637 was significantly altered by PP2A inhibitors (MC‐LR) and agonists (FTY720) (Figure S6B, Supporting Information). Further, increased phosphorylation of ULK1 at the Ser 637 site was further verified in BMMs and RAW264.7 after PP2Ac knockdown by western blot (Figure S6C,D, Supporting Information) and immunofluorescence staining of ULK1 (Figure S6E,F, Supporting Information). To further determine the ULK1‐dependent role of PP2Ac in autophagy regulation, ULK1 was blocked after PP2Ac knockdown in BMMs or RAW264.7. We found that ULK1 inhibition partially reversed the dephosphorylation level of ULK1 after PP2Ac was knocked down by western blot assay (Figure 4C,D) and immunofluorescence staining (Figure 4E,F). To clarify the regulation of autophagy by PP2Ac through ULK1, we performed western blot of LC3II/I and Beclin1 (Figure 4G,H), TEM observation (Figure 4I,J), and mCherry‐GFP‐LC3 staining (Figure 4K,L) in BMMs and RAW264.7. Similarly, the autophagy level was rescued via ULK1 inhibition. We then determined the role of ULK1 in PP2Ac‐regulated osteoclastogenesis and found that ULK1 inhibition facilitated the osteoclastogenesis process and enhanced osteoclast function after PP2Ac knockdown (Figure S6G–N, Supporting Information), indicating that PP2Ac‐mediated osteoclastogenesis is dependent on ULK1.

### 2.6 PP2Ac Regulates ULK1 through Direct Binding Interaction

**Figure 5:**
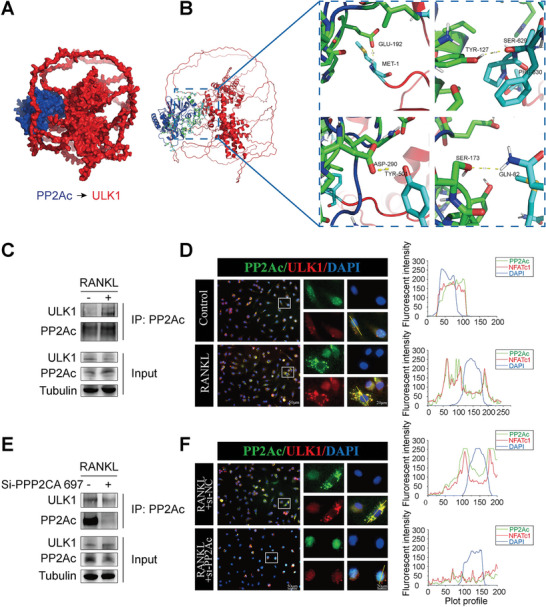
PP2Ac directly activates ULK1 to mediate autophagy. A) Molecular docking spherical graphs indicating that PP2Ac was surrounded by ULK1 molecules. B) Cartoon and stick graph showing that PP2Ac and ULK1 could be interconnected with several residues via by hydrogen bonds. C) Co‐immunoprecipitation assay to detect the interaction between PP2Ac and ULK1 in BMMs with RANKL induction. The co‐precipitated ULK1 was subsequently detected by western blot analysis. D) Immunofluorescence co‐localization and fluorescence intensity were used to observe the spatial location of PP2Ac and ULK1 in the absence and presence of RANKL‐induced BMMs. E) PP2Ac and ULK1 interaction determined by co‐immunoprecipitation and western blot in RANKL‐induced BMMs by PP2Ac intervention with or without siRNA knockdown. F) PP2Ac and ULK1 spatial positioning and fluorescence intensity were examined with immunofluorescence co‐localization staining with or without siRNA PP2Ac knockdown in RANKL‐induced BMMs.

To study the potential direct binding between PP2Ac and ULK1, molecular docking simulation results revealed that ULK1 completely enveloped the structure of PP2Ac (**Figure** **5**A), and through hydrogen bonds, frequent interactions occurred between the Glu‐192, Tyr‐127, Asp‐290, and Ser‐173 residues of PP2Ac with ULK1 (Figure 5B). To further confirm the interaction between PP2Ac and ULK1, Co‐IP experiments were conducted which demonstrated that RANKL‐induced BMMs owned a higher binding affinity between PP2Ac and ULK1 than the non‐induced group (Figure 5C). Knockdown of PP2Ac decreased the expression of PP2Ac and significantly attenuated the binding between PP2Ac and ULK1 in RANKL‐induced BMMs (Figure 5D). Furthermore, to analyze the spatial location and positional relationship between PP2Ac and ULK1, immunofluorescence co‐localization and immunofluorescence intensity were selected. Likewise, significant co‐localization of PP2Ac and ULK1 in RANKL‐induced BMMs was observed (Figure 5E), and knockdown of PP2Ac resulted in decreased co‐localized fluorescence intensity of PP2Ac and ULK1 (Figure 5F). This evidence suggests that PP2Ac directly binds ULK1, thereby providing a favorable condition for ULK1 dephosphorylation to regulate autophagy.

### 2.7 The mTORC1 Involved in PP2Ac‐Regulated Autophagy

Maintaining the balance between phosphorylation and dephosphorylation is essential for cellular homeostasis. We considered that PP2Ac might be regulated by upstream molecules to fine‐tune the balance of ULK1 phosphorylation. The mammalian target of rapamycin (mTOR) acts as a critical serine/threonine kinase that regulates cell proliferation and growth in mammals.^[^ ^15^ ^]^ Within the mTOR complex, mTORC1, as the primary target of rapamycin, is best characterized by autophagy regulation.^[^ ^16^ ^]^ Previous studies have identified that mTORC1 is crucial for autophagosome initiation and formation inhibition by phosphorylating ULK1.^[^ ^17^ ^]^ Our current study suggests that rapamycin, an inhibitor of the mTORC1 molecule, promotes autophagy during osteoclastogenesis, and KEGG analysis also suggests the involvement of the mTOR pathway. Therefore, we speculated that mTORC1 might be involved in autophagy regulation, acting as a regulatory molecule upstream of PP2Ac and thus subtly regulating the phosphorylation balance of ULK1.

The phosphorylation level of mTORC1 in RANKL‐induced BMMs was examined initially, and the western bolt assay indicated the mTORC1 phosphorylation level reduced upon RANKL induction (Figure S7A, Supporting Information). Treatment with RAPA further synergized the effect of RANKL to inactivate the phosphorylation level of mTORC1. To clarify the regulatory relationship between mTORC1 and PP2Ac, we also examined the expression of PP2Ac in the presence of RAPA and found that mTORC1 inhibition increased PP2Ac expression compared to RANKL induction alone (Figure S7B, Supporting Information). These results suggest that mTORC1 inhibition could assist PP2Ac‐regulated autophagy during osteoclastogenesis. We further explored the effect of mTORC1 inhibition on ULK1 phosphorylation levels by blocking PP2Ac expression after mTORC1 inhibition and found that PP2Ac inhibition partially reversed the rapamycin‐induced phosphorylation of ULK1, indicating that mTORC1 participates in the regulation of ULK1 phosphorylation through PP2Ac. Immunofluorescence experiments further confirmed the changes in ULK1 intensity (Figure S7C, Supporting Information).

To study whether mTORC1 is involved in the regulation of autophagy during osteoclastogenesis, RAPA was added to RANKL‐induced BMMs. Western blot results revealed higher expression levels of LC3II/I and Beclin1 compared to the RANKL‐induced BMMs group (Figure S7D, Supporting Information). Conversely, the enhanced autophagy was partially reversed with the PP2Ac inhibitor during osteoclastogenesis. The GFP‐mCherry‐LC3 experiment and TEM test further confirmed the aforementioned autophagy changes (Figure S7E,F, Supporting Information). It shows that mTORC1 participates in regulating the phosphorylation balance of ULK1 by mediating PP2Ac.

### 2.8 Inhibition of PP2Ac Ameliorated OA Progression

**Figure 6:**
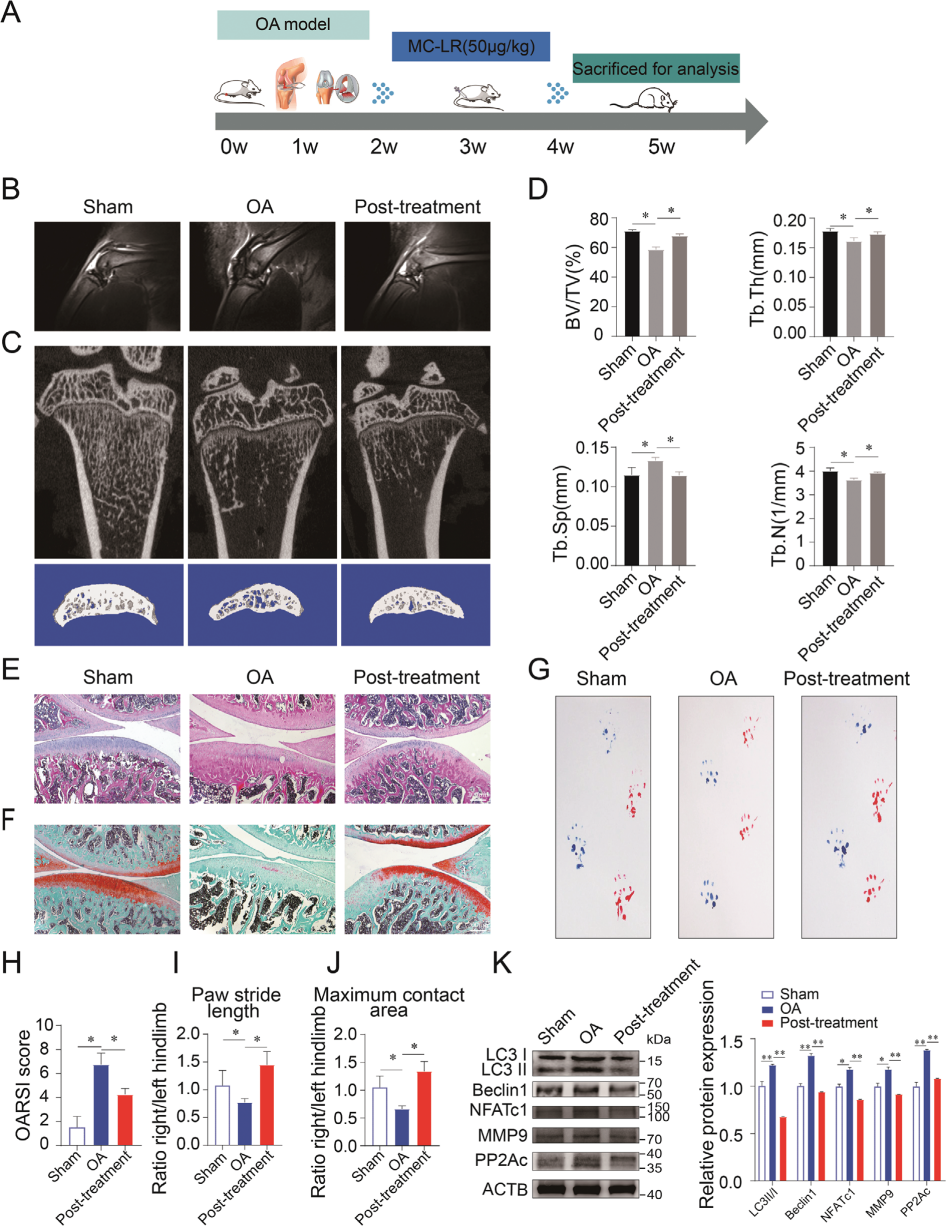
Treatment with PP2Ac inhibitor alleviates the progression of early OA. A) Flow chart to illustrate the timeline of the OA model induced in rats by ACLT surgery and the subsequent intervention with a PP2Ac inhibitor. B) The T2‐weighted MRI images were used to detect the signal changes in knee cartilage and subchondral bone tissue of rats with OA models as well as the treatment group. C) Micro‐CT detection and 3D reconstruction observation (coronal and sagittal position) of subchondral bone trabecular bone in the OA model group and treatment group. D) The trabecular bone parameters, including volume fraction (BV/TV), trabecular number (Tb. N), trabecular thickness (Tb. Th), and trabecular separation (Tb. Sp), were quantitatively assessed and compared among the three groups. E) H&E staining showing the histological changes following PP2Ac inhibition. F) Safranin O fast green staining was used to observe the morphology and lesions of the articular cartilage and subchondral bone trabeculae. G) Observational gait analysis to assess knee joint function recovery of rats 4 weeks after ACLT surgery. The right hind foot of the OA model group was marked in red ink, while the sham group was marked in blue ink. H) The severity of knee arthritis injuries was assessed by the OARSI score based on Safranin O fast green staining. I, J) The ink blotting trial images measured the distance and area of hind foot blotting, and these parameters were quantitatively evaluated. K) Western bolt assay and gray value quantitative analysis detected the expression of osteoclasts, autophagy markers, and PP2Ac in the subchondral bone of rats with pharmacological inhibition of PP2Ac. (*n* = 5, mean ± SD; *p < 0.05; **p < 0.01; versus sham group).

To explore the intervention of PP2Ac on early OA, a 2‐week OA model in rats was selected, followed by intraperitoneal injection of MC‐LR (50ug kg^−1^ bw) for 2 weeks (**Figure** **6**A), and no significant signs of toxicity were observed in rats during the experimental period (Figure S8, Supporting Information). First, we found that compared with the OA group, subchondral bone marrow edema in the treatment group was alleviated and the damaged signal of subchondral bone was weakened by MRI detection (Figure 6B). Micro‐CT detection showed that PP2Ac inhibition promoted trabecular bone number in the coronal plane and increased tibial subchondral bone volume in the sagittal plane (Figure 6C). Trabecular bone parameters, such as BV/TV, Tb/Th, Tb/Sp, and Tb/N, also indicated an improvement in early OA after PP2Ac inhibition (Figure 6D). H&E staining, safranine O fast green staining, and OARSI score analysis implied an improvement in the roughness of the articular surface cartilage, palliation of cartilage degeneration, and a decrease of OARSI score with PP2Ac inhibition (Figure 6E,F,H). Gait analysis revealed the moving distance and contact area ratio of the hind paw in the treatment group were higher than those in the OA group, signifying that PP2Ac inhibition could postpone the progression of OA (Figure 6G,I,J). TRAP staining showed a lower number of positive osteoclasts in the subchondral bone of the treatment group compared to the OA group (Figure S9A, Supporting Information). Immunofluorescence co‐staining of NFATc1 and PP2Ac also indicated that inhibiting PP2Ac could suppress functional gene expression of osteoclasts (Figure S9B, Supporting Information). Western blot assay showed that inhibiting PP2Ac could also lower the levels of osteoclasts and autophagy (Figure 6K). These findings suggest the therapeutic effect of inhibiting PP2Ac on OA.

### 2.9 Inhibition of PP2Ac Prevented OA Development

To investigate the preventive effect of PP2Ac inhibitors on OA, rats were initially injected with MC‐LR (50ug kg^−1^ bw) for two weeks, as shown in Figure S10A (Supporting Information), followed by inducing an OA model for the next two weeks. Compared to the OA model, MRI detection suggested a decreased injury edema signal at the subchondral bone with PP2Ac inhibitor (Figure S10B, Supporting Information). We also found weakened trabecular bone aggregation with trabecular parameter analysis (Figure S10C,D, Supporting Information). H&E staining revealed the uneven articular surface improved after PP2Ac inhibition (Figure S10E, Supporting Information). Furthermore, with safranine O fast green staining, we found reduced articular glycans and increased shallow cartilage layer, and decreased OARSI score in the treatment group (Figure S10F,H, Supporting Information). Gait observation indicated that the left posterior/right posterior paw print area ratio and ipsilateral stride length were improved in the pre‐treatment group, implying PP2Ac inhibition could improve behavioral function and prevent OA progress (Figure S10G,I,J, Supporting Information). TRAP staining revealed fewer positive osteoclast cell numbers in subchondral bone when pre‐intervened with PP2Ac inhibitor (Figure S11A, Supporting Information). Similarly, the weaker expression intensity of osteoclasts and autophagy level was shown by immunofluorescence staining with PP2Ac inhibition (Figure S11B, Supporting Information). Western blot experiments showed that early inhibition of PP2Ac expression could also decrease the expression of osteoclast markers and autophagy levels in the early stage of OA (Figure S10K, Supporting Information), which indicated the preventive effect of pre‐applied PP2Ac inhibition on OA.

## 3 Discussion

Our study revealed that inhibiting PP2Ac suppressed osteoclastogenesis in the early stage of OA by regulating autophagy through ULK1. To elucidate this effect, osteoclast precursor cells were selected and induced with RANKL by knocking down or inhibiting PP2Ac expression and ULK1 could be involved in osteoclastogenesis through directly binding to PP2Ac. Additionally, mTORC1 inhibition enhanced PP2Ac activation and regulated autophagy during osteoclastogenesis. Furthermore, our in vivo intervention with PP2Ac inhibitors prevented OA exacerbation and delayed OA progression. Collectively, our findings highlighted that PP2Ac may represent a promising therapeutic target for modulating autophagy in OA.

Abnormal osteoclastogenesis in the subchondral bone of OA initiates an increased rate of bone remodeling, resulting in reduced subchondral bone plate thickness.^[^ ^18^ ^]^ Our previous work showed that inhibiting PP2A weakened RANKL‐induced osteoclastogenesis and alleviated osteoclastic resorption in peri‐prosthetic osteolysis.^[^ ^12^ ^]^ However, how PP2A regulation affects osteoclastogenesis is yet to be elucidated. In our study, we observed that PP2Ac could regulate ULK1 phosphorylation via the autophagy pathway to mediate osteoclastogenesis. The dephosphorylation of ULK1 by PP2A promoted its activation under starvation conditions.^[^ ^14^, ^19^ ^]^ Our study found that OCPs were not exposed to starvation, but instead induced by RANKL, and the PP2A catalytic subunit also participates in the dephosphorylation of ULK1 to promote autophagy. Meantime, the knockdown of PP2Ac weakened the dephosphorylation of ULK1 during osteoclastogenesis. Previous studies have suggested that ULK1 has different phosphorylation sites that could inhibit or activate autophagy.^[^ ^20^ ^]^ Our current research showed the phosphorylation activity of ULK1 decreased with the high expression of PP2Ac, and we speculated that phosphorylated ULK1 inhibits autophagy. We proposed that ULK1 might have one or several phosphorylation sites that could bind to PP2Ac to regulate autophagy. We then screened possible phosphorylation sites, including Ser 556, Ser 757, and Ser 637 for validation.^[^ ^14^, ^21^ ^]^ And then, the reduction of PP2Ac expression attenuated the dephosphorylation of ULK1 at the S637 site. This regulatory association is consistent with Wong PM et al with pancreatic ductal adenocarcinoma cells in vitro.^[^ ^14a^ ^]^ That is, in addition to the PP2A holoenzyme, high expression of PP2Ac alone in vitro is sufficient to dephosphorylate ULK1. Therefore, our findings could help elucidate the ULK1 involved in the regulation of the underlying mechanism of PP2Ac‐mediated autophagy during osteoclastogenesis.

PP2A is mainly responsible for post‐translational dephosphorylation and is essential for regulating cell metabolism, proliferation, and apoptosis.^[^ ^22^ ^]^ Previous studies have suggested the regulation of PP2A activity during autophagosome formation is complicated and multifaceted.^[^ ^23^ ^]^ Our present study found rapamycin, as mTORC1 inhibitor, not only positively regulates autophagy but increases the expression of PP2Ac during osteoclastogenesis. Many studies have shown that mTOR acts as a repressor of PP2A activity and that mTOR inhibition could release PP2A activity.^[^ ^24^ ^]^ Alpha4 integrin is a common regulator of PP2A phosphatase activity in mammals.^[^ ^25^ ^]^ Under nutrient‐rich conditions, mTOR phosphorylates alpha4, promoting the formation of the alpha4‐PP2A (including catalytic subunit) complex that sustains the phosphatase enzyme in an inactive state. However, under starvation conditions, once mTOR signaling is suppressed, the PP2A catalytic subunit is released from inhibitory protein alpha4, and ultimately, leading to phosphatase activation.^[^ ^26^ ^]^ Consistent with previous observations, starvation triggers the activation of PP2Ac, which rapidly dephosphorylates ULK1 and induces autophagy. Conversely, the recombinant alpha4‐PP2A complex could not dephosphorylate ULK1.^[^ ^20^ ^]^ This evidence suggested that mTOR has a negative regulatory relationship with PP2Ac. Intriguingly, several studies suggested mTOR and PP2A interact through a reciprocal regulatory network to regulate biological processes, and PP2A also could dephosphorylate mTOR and its downstream effectors (4EBP1 and p70S6K) to inhibit mTOR activity in cancer diseases.^[^ ^27^ ^]^ Meanwhile, some studies have suggested that mTORC1, via phosphorylated function of kinases, could participate in the regulation of ULK1 during autophagosome biogenesis.^[^ ^14^, ^28^ ^]^ In our study, we have preliminarily explored the regulatory relationship between mTORC1 and PP2Ac‐mediated autophagy during osteoclastogenesis, and we speculate that mTORC1 may affect the activity of PP2Ac or provide a phospho‐switch with PP2Ac to balance the coordinated phosphorylation of ULK1 to regulate autophagy. Further research is needed to explore more attractive mechanisms regarding the potential feedback loop between mTORC1 and PP2Ac.

Our current research focuses on the osteoclast aspect of osteoarthritis in subchondral bone, and the single‐cell gene dataset also reminds us that other cell types may play a role in OA. Among these, cartilage damage is an important feature of this disease. Currently, researchers are investigating the role of PP2A in chondrocytes. In studies of the chondrocyte cell cycle, fibroblast growth factor (FGF) induced chondrocyte growth arrest is a unique cell type‐specific response. Knockdown of PP2A, a universally expressed multisubunit phosphatase with a B55α regulatory subunit, prevented p107 dephosphorylation and chondrocyte growth arrest. This mechanism may be a general signaling pathway by which PP2A initiates cell cycle arrest when required by external signals.^[^ ^29^ ^]^ In chondrocyte hypertrophy, parathyroid hormone‐related peptide (PTHrP) inhibits chondrocyte hypertrophy by inhibiting Runx2 transcription through activation of PP2A to dephosphorylate HDAC4.^[^ ^30^ ^]^ It has also been found by Ali A et al. in osteoarthritic diseases that licorice regulates HDAC4 activation through PTH1R/PKA/PP2A signaling pathway and SOX9/β‐catenin signaling pathway to inhibit IL‐1β‐induced chondrocyte hypertrophy.^[^ ^31^ ^]^ In the context of chondrocyte differentiation, it has been reported that mesenchymal stem cells (MSCs) require stringent regulation of gene expression for cartilage formation. The expression of octamer‐binding transcription factor 4 (Oct4) was found to be positively correlated with CIP2A, a natural inhibitor of PP2A. Overexpression of CIP2A in Oct4‐deficient MSCs enhanced cartilage matrix deposition and the expression of Col2a1, Col10a1, Acan, and Sox9. These results suggest that Oct4 acts as an important regulator of chondrogenesis, in part through the CIP2A/PP2A signaling pathway.^[^ ^32^ ^]^ These studies indicated that PP2A and its subunits may be involved in regulating chondrocyte proliferation, differentiation, and hypertrophy, and play a certain role in regulating chondrocytes. To macrophage polarization, genetic deletion of the SE translocation (SET) in myeloid cells inhibits the entry of tumor‐associated macrophages (TAMs) into hypoxic tumor regions. Mechanistically, cytoplasmic retention of SET, through inhibition of PP2A, increases ERK and P38 pathways, which facilitates the movement of TAMs into hypoxic regions, migration, and polarization toward the M2 phenotype. Thus, PP2A may also be involved in tumor immunity by regulating macrophage localization and function in tumors.^[^ ^33^ ^]^ Additionally, PP2A‐SET interactions have been found to induce granzyme B expression and modulate human natural killer (NK) cell cytotoxicity.^[^ ^34^ ^]^ Although PP2A can participate in regulatory roles in various cellular subpopulations, its subunits, including PP2Ac, have received little attention in OA, particularly in osteoclast studies. Further research is needed to investigate their role in different cellular subpopulations.

In summary, our study exhibited that PP2Ac inhibition decreased autophagy during osteoclastogenesis and ameliorated the destruction of early OA. This research sheds light on the post‐translational molecular mechanisms underlying PP2Ac‐mediated autophagy and suggests that a single phosphatase subunit could play diverse biological roles in human diseases. Our current findings indicate that targeting PP2Ac could be a valuable and attractive therapeutic strategy for OA treatment. Further investigations were needed to fully comprehend the exquisite and complex regulatory mechanisms of the PP2Ac‐mediated autophagy network.

## 4 Experimental Section

### Reagents and Antibodies

PP2A inhibitor Microcystin‐LR (MC‐LR), PP2A activators Fingolimod hydrochloride (FTY720), autophagy inhibitors 3‐Methyladenine (3‐MA), and activators rapamycin (RAPA), ULK1 inhibitor (SBI‐0206965) were purchased from MCE (MedChemExpress, Shanghai, China). Recombinant recombinant rat M‐CSF and RANKL were purchased from PeproTech (London, UK), and RANKL reagent for murine cell lines was acquired from the R&D Company (Minneapolis, USA). Primary antibodies used in western blot and Co‐IP assay are as follows: Rabbit anti‐PP2Ac (1:1000, ab32104, Abcam), Rabbit anti‐PP2Ac (1:1000, ab32065, Abcam), Rabbit anti‐ULK1 antibody (1:1000, ab167139, Abcam), Rabbit anti‐phos‐ULK1 (S638; 1:1000, PA5‐105129, Thermo Fisher Scientific), corresponding to S637 sites of ULK1 in Rats. Rabbit anti‐phos‐ULK1 (S757; 1:1000, ab229909, Abcam), Rabbit anti‐phos‐ULK1 (S556; 1:500, ab203207, Abcam), Rabbit anti‐LC3 (1:1000, ab192890, Abcam), Rabbit anti‐Beclin 1 (1:1000, ab207612, Abcam), Rabbit anti‐mTORC1 (1:1000, ab25880, Abcam), Rabbit anti‐NFATc1 (1:1000, A1539, ABclonal), Rabbit anti‐MMP9 (1:1000, A2095, ABclonal), Rabbit anti‐ACTB (1:1000, ab8227, Abcam), Mouse anti‐Tubulin(1:1000, ab28035, Abcam). HRP‐conjugated Goat anti‐Rabbit and Goat anti‐Mouse antibodies as secondary antibodies and normal rat IgG were obtained from Beyotime Institute of Biotechnology (Shanghai, China). Protein A/G Magnetic Beads were acquired from MCE Company. Fetal bovine serum (FBS) and dulbecco's modified eagle's medium (DMEM) were purchased from Hyclone Company.

### Bioinformatics Analysis

The GEO Database (https://www.ncbi.nlm.nih.gov/geo/) was selected to search and screen high throughput sequencing datasets with the keys word “osteoarthritis” or “OA” and “single cell” or “scRNA” within the organism of Homo sapiens. Through the detection of the GEO database, GSE196678 and GSE104782 were single‐cell sequencing data related to OA. The GSE104782 dataset was excluded mainly because only chondrocyte types were involved. The GSE196678 dataset involves four subchondral bone OA samples, contains a variety of cell subsets, and meets the requirements. The high throughput scRNA sequencing dataset was from the GPL24676 Illumina NovaSeq 6000 (Homo sapiens) platforms. Then the selection and screening of the gene datasets containing these keywords “osteoclastogenesis” or “osteoclasts” or “BMMs” and “M‐CSF” or “RANKL” was continued and with the following criteria: 1) reliable source with detailed information; 2) expression profiling by high throughput sequencing or array; 3) transcriptome analysis. Based on the filtering criteria mentioned above, the GSE176265 was selected for analysis. Next, the BMMs treated with M‐CSF and RANKL were compared for 0, 24, 48, and 72 h. The microarray dataset was from the following platforms: GPL6887: Illumina MouseWG‐6 v2.0 expression beadchip.

For scRNA‐seq analysis methods, the GSE196678 datasets for each individual sample were downloaded and processed using the “Seurat” package (version 4.0; http://satijalab.org/seurat/) using R software (version 4.2).^[^ ^35^ ^]^ The following filter conditions were set: nFeature_RNA > 300 and nFeature_RNA < 7000, and percent.mt < 10, and percent.HB < 3, and then nCount_RNA < 100 000. Further bioinformatics analysis was conducted using a total of 26750 filtered cells. Then ScaleData and NormalizeData were selected to standardize and homogenize the data, and 3000 highly variable genes (HVGs) for subsequent PCA dimension reduction and principal component extraction with the FindVariableFeatures function. The Cluster analysis was performed by FindNeighbors and the FindClusters functions embedded in the Seurat package. Identified clusters were visualized on 2D maps generated using t‐SNE methods. To annotate cell clusters, the SingleR package was used to annotate cell subpopulations with the human reference genome. DEGs (adjusted *p*‐val < 0.05) were identified with high discriminative power between groups using the FindAllMarkers function in Seurat, which employed a default nonparametric Wilcoxon rank sum test and Bonferroni correction.

NetworkAnalyst 3.0 (https://www.networkanalyst.ca/), an online tool, was used for comprehensive gene expression profiling analysis and network visualization in the bulk transcriptome bioinformatics analysis of both GSE176265 and our own RNA‐Seq data.^[^ ^36^ ^]^ The DEGs were identified using the Limma statistical method with absolute log2(fold change) ≥ 1 and adjusted *p*‐value < 0.05 as threshold values.

Gene set enrichment analysis (GSEA) was performed with GSEA software (v4.1.0, https://www.broadlnstitute.org/gsea/) to determine significant differences in the set of genes expressed between groups regarding phenotypic changes.^[^ ^37^ ^]^ Briefly, the regularized log‐normalized counts were compared from the transcriptome data (GSE176265) with the up‐ or down‐regulated gene sets. For each analysis, gene set permutations were set 1000 times, and the parameters of normalized enrichment score (NES) and nominal *p*‐value were considered the statistical significance and enrichment degree. All other settings were left as default.

Metascape (http://metascape.org) is a public database website used to analyze the primary function of gene sets through gene function enrichment and classification.^[^ ^38^ ^]^ The gene functions and interconnections are annotated using gene ontology (GO) and the pathway annotation is mapped using the Kyoto Encyclopedia of Genes and Genomes (KEGG). A cutoff value of *p* < 0.05 was set. The results with a minimum enrichment name of at least 3 and an enrichment factor of > 3.0 were collected and grouped into clusters.

### Human Tissue Samples

The human knee joints were obtained from the First Affiliated Hospital of Soochow University with institutional review board approval (2022‐No.224). A total of 12 fresh tibial plateaus were collected, with knee joints from young patients who underwent amputation due to traffic accidents as the control group (four cases), and the medial tibial plateau after unicompartmental knee arthroplasty as the observation group for OA (eight cases), and clinical information on the human samples was provided in Table S1 (Supporting Information). Under sterile conditions, a 12 mm diameter trephine was selected to acquire the cartilage‐subchondral bone composite tissues perpendicular to the articular cartilage surface, with dimensions of ≈12 × 12 × 10 mm (length × width × height). All specimens were prepared for subsequent study.

### The Rat OA Model

90 healthy male SD rats (Sprague Dawley, 8 weeks old) were provided by the Experimental Animal Center of Soochow University. In accordance with the guidelines of the Care and Use of Laboratory Animals, all animal procedures and experiments were conducted and approved by the Animal Ethics Committee of Soochow University (SUDA20220713A02). The OA model was induced by performing the anterior cruciate ligament transection (ACLT) procedure. Briefly, the rats were prepared for the operation by skin preparation, weighing, and intraperitoneal anesthesia with 2% sodium pentobarbital (30 mg kg^−1^) before the operation. After a sterile drape, the right knee joint was incised to expose the medial side of the patella, and the joint bundle was cut along the medial side of the patellar tendon. The lateral patella was displaced, and the knee joint was transected with micro‐scissors under full flexion. The left knee served as a control group and underwent the same procedure, except that the anterior cruciate ligament was not severed. Antibiotics and analgesics were injected after the surgery for three consecutive days to prevent postoperative infection and relieve pain. MC‐LR was dissolved in sterile saline and injected intraperitoneally into the rats every three days for a 2‐week period.

### CCK8 Assay

To estimate cell growth and viability of BMMs, CCK8 cell assays were performed under the manufacturer's instructions (Beyotime Institute of Biotechnology, China). 1 × 10^4^ cells per well were cultured in a 96‐well plate and various concentrations of reagents were treated with BMMs for ≈48 h. Subsequently, each well was incubated with the CCK8 reagent for 2 h, after which the OD value was recorded at 450 nm by a microplate reader (BioTec Instruments, Inc. USA).

### Cell Culture

In order to isolate and culture bone marrow‐derived macrophages (BMMs), 4–6‐week‐old male SD rats were euthanized with cervical dislocation after anesthesia, and after sterilization with 75% ethanol, the rats were placed on a sterile glass plate. The femur and tibia were completely isolated, respectively, and the intact bones soaked in PBS were transferred to an ultra‐clean workbench. Cut off metaphyses of the femur and tibia respectively to fully expose the medullary cavity, the diaphysis cavities were flushed with a complete medium containing 30 ng mL^−1^ M‐CSF with a 10 mL sterile syringe using a 20‐gauge needle repeatedly until the medullary cavity turns white. Resuspended with red blood cell lysate, after lysing and centrifuging cells, the obtained cells were cultured for 16 h with a 10 cm petri dish. The supernatant was then transferred and treated with M‐CSF (30 ng mL^−1^) for 3 days. The adherent cells obtained were considered osteoclast precursor cells (OCPs) and BMMs were further cultured with M‐CSF (30 ng mL^−1^) and RANKL (50 ng mL^−1^) for the following experiments.

Raw264.7 derived from a mouse macrophage cell line was obtained from Shanghai Cell Bank (Shanghai, China) and expanded in high‐glucose DMEM with 10% bovine serum. The cells used in the experiment were selected from passages 2 to 15, treated as OCP, and then induced into osteoclasts with RANKL (50 ng mL^−1^).

### MRI Detection

To observe structural signaling changes in OA, live animal magnetic resonance imaging (MRI) was performed on rats. The rats were anesthetized, and real‐time respiratory monitoring was conducted during imaging. All MRI images were obtained via a 3T Siemens Magnetom Prisma machine (Siemens, Erlangen, Germany) with customized small animal imaging coils. The MRI acquisition parameters for the T2‐weighted imaging spin‐echo (SE) sequence were set with the following parameters, repetition time (TR) was 3000 ms, the echo time (TE) was 83 ms, and the field of view (FOV) was selected as 70 × 70 mm with a slice thickness of 1.5 mm per layer. MRI evaluation of rat knee joints requires at least two staff members.

### Micro‐CT Analysis

To evaluate and observe morphological features of subchondral trabecular bone, the rat bilateral knee joint samples were first fixed with 4% paraformaldehyde. The samples were subsequently scanned and analyzed by the high‐resolution SkyScan1076 micro‐CT machine. (Bruker, Aartselaar, Belgium). The scanning parameters for the following analysis were set as follows: the scanning resolution was 18 µm per layer, the rotation angle was 180°, and the rotation step was set by 0.6° with 1.0 mm Al filtration. The regions of interest (ROIs) at the subchondral bone trabecular area within the same position were selected to collect data, then 2D and 3D image reconstruction were performed using 3D‐MED image processing software, and trabecular bone microstructure was quantitatively analyzed, such as bone volume over total volume (BV/TV), trabecular number (Tb.N), trabecular thickness (Tb.Th), trabecular separation/spacing (Tb.Sp), and the total volume of osteophytes.

### Adenovirus Transfection (Ad‐mCherry‐GFP‐LC3 Staining)

To observe autophagic alterations in BMMs and RAW264.7 during osteoclastogenesis, BMMs (3 × 10^5^ cells per well) and RAW264.7 (1 × 10^5^ cells per well) were separately seeded in 24‐well plates. Cells were transfected at an MOI of 20, then changed into fresh culture medium after 24 h, and continued to be cultured for the following 24 h. Under the observation of a fluorescence microscope, when autophagy occurred, the mCherry‐GFP‐LC3 fusion protein was transferred to the autophagosome membrane, forming yellow fluorescent punctae. When the fusion of lysosomes and autophagosomes forms autophagolysosomes, the acidic environment quenches GFP fluorescence, while mCherry fluorescence was not affected, and autophagolysosomes exhibited red fluorescence. The intensity of autophagic flow could be evaluated by counting spots of different colors. After that, the culture was treated with M‐CSF and RANKL solutions and incubated for 16 h. Autophagy was then observed using a fluorescence microscope (Zeiss, Germany).

### TEM Observation

To detect autophagosomes during osteoclastogenesis, transmission electron microscopy (TEM) was selected. Briefly, BMMs (1 × 10^6^ cells per well) and RAW264.7 (5 × 10^5^ cells per well) were separately seeded in a six‐well plate and incubated for 16  h with RANKL. OCPs were collected and fixed with 2.5% glutaraldehyde. Then rinsed, dehydrated, solidified, sectioned, stained, and finally observed the number of autophagosomes in different groups with a TEM machine (FEI Tecnai Spirit, USA).

### TRAP Staining

To induce osteoclastogenesis in vitro, BMMs (2 × 10^4^ cells per well) and RAW264.7 cells (1 × 10^4^ cells per well) were separately seeded into 48‐well plates and incubated with or without M‐CSF (30 ng mL^−1^) and RANKL (50 ng mL^−1^) for 4–5 days. TRAP staining (Bizhong Biotechnology Co., Ltd.Suzhou.China) was performed in accordance with the kit instructions: after fixation with 4% paraformaldehyde for 20 min, the staining solution I was added to the incubator at 37 °C for 30 min in the dark. Then washing cells twice with PBS, nuclei were counterstained with reagents III and IV for 30 min. Images were taken and saved using an inverted microscope (Carl Zeiss, Germany). The wine‐red TRAP‐positive multinucleated cells with three or more nuclei were viewed as mature osteoclasts and counted per field (*n* = 5).

### Bone Resorption Assay and Scan Electron Microscopy (SEM) observation

To obverse the function of osteoclasts, bone resorption pits were assessed. Sterilized bone slices (Shanghai Yaoang Biotechnology, China.) were flated in a 96‐well plate. BMMs (5 × 10^3^ cells per well) and RAW264.7 (2 × 10^3^ cells per well) were incubated with complete medium supplemented using M‐CSF (30 ng mL^−1^) and RANKL (50 ng mL^−1^). After induction for 7 days, bone slices were ultrasonically cleaned and dried and observed with FEI Quanta 250 scanning electron microscope (Hillsboro, USA). Bone resorption pit area measurements were performed with Image J 1.8.0 software (NIH, USA).

### Western Blot Assay

To detect cellular protein expression, western blot was used according to standard protocol with corresponding antibodies. Briefly, BMMs (1.5 × 10^6^ cells per well) and RAW264.7 cells (1 × 10^6^ cells per well) were separately seeded in six‐well plates overnight. For autophagy protein isolation, these cells were stimulated with RANKL to form pre‐osteoclasts for 16 h. To extract osteoclast‐specific proteins, the BMMs or RAW264.7 cells line were continuously cultured for the other 4 days. Afterward, cells were collected and lysed to extract proteins, and BCA kits (New Cell and Molecular Biotech, China) were used to detect the protein concentration. Protein sample electrophoretic separation was performed on 6–12% SDS‐PAGE electrophoresis gel. The bands were subsequently transferred to the NC membrane (Beyotime Institute of Biotechnology, China), blocked for 1 h at room temperature, and treated by the primary antibody and specific species HRP‐labeled secondary antibody (Abcam). The bands were exposed to a gel imaging system using the ultra ECL chemiluminescence kit (New Cell and Molecular Biotech, China), and the grayscale values of the protein bands with Image J software were analyzed.

### Molecular Docking

Molecular docking predicts the binding interaction between ligand molecules and target proteins, providing insight into binding sites. UniProt Knowledgebase (https://www.uniprot.org/), as a hub of integrated protein data, was selected to download 3D structures of molecules.^[^ ^39^ ^]^ The UniProt identifiers of Ppp2ca, Ppp2cb, and ULK1 proteins from rattus norvegicus species were downloaded (P63331, P62716, and D3ZMG0, respectively). HADDOCK (https://wenmr.science.uu.nl/haddock2.4/), as an online protein–protein docking website that relied on biochemical or biophysical information,^[^ ^40^ ^]^ performed protein docking of PP2Ac and ULK1 molecules. A total of ten independent runs were chosen for each structure. The input parameters were selected by default and screened possible surface residues around active residues within a radius of 6.5 Å. Docking parameters included distance restraints, clustering parameters, dihedral and hydrogen bonds restraints, symmetry restraints, and energy and interaction parameters, also set to default. Molecular graphics were visualized using PyMOL software (http://www.pymol.org), and automated docking of protein molecules was achieved through the InterfaceResidues code (https://pymolwiki.org/index.php/InterfaceResidues).

### Co‐Immunoprecipitation (Co‐IP) Experiment

To explore protein interaction, BMMs were seeded into six‐well plates at a cell density of 2 × 10^6^ per well and then incubated with RANKL for 16 h. After centrifugation, BMMs were collected and lysed on ice using 120 µL of immunoprecipitation lysis buffer mixed with 2 µL of protease inhibitor. (Beyotime Institute of Biotechnology, China). The supernatant was harvested, and the protein concentration was measured using a BCA kit. The primary antibody of 1 µg and 30 µL Protein A/G Magnetic Beads (MedChemExpress, USA) were transferred to the cell lysate and incubated at 4 °C overnight with gentle shaking. After the beads were centrifuged to the bottom and removed the supernatant was carefully washed three times by 500 µL lysis buffer. The final samples were mixed with SDS buffer and then boiled for denaturation. Western blot assay was performed as described above.

### Immunofluorescence Staining and Colocalization Assay

After washing with PBS, BMMs and RAW264.7 cells were fixed with 4% paraformaldehyde for 20 min, followed by permeabilization with 0.1% Triton X‐100 for 10 min. Cells were then blocked with 5% bovine serum albumin (BSA) for 30 min at room temperature. Subsequently, different species primary antibodies were added overnight, followed by incubation with the corresponding secondary antibodies (Alexa Fluor 647‐conjugated anti‐rabbit IgG antibody or Alexa Fluor 488‐conjugated anti‐mouse IgG antibody) for 1 h at room temperature. All samples were counterstained by DAPI for 5 min and photographed with an Axiovert 40C optical microscope (Zeiss, Germany).

Co‐localization evaluations in BMMs and RAW264.7 cells were conducted by double immunofluorescence staining. Briefly, 2D scattergrams of each image were evaluated for signal spatial colocalization and overlap coefficient according to Manders (MOC) Pearson's correlation coefficient (PCC) and were calculated from pixels with intensity values. With the help of the Co‐localization Analysis ImageJ plugin, multichannel images were analyzed and automatically quantification. And data visualization of co‐localized fluorescent signals was finished with Origin 2018 software (Northampton, USA).

### Histological Staining, TRAP, and Immunohistochemical Staining

To observe the histopathological changes in the knee joint, the samples were fixed, decalcified, dehydrated, embedded, and sectioned into 6 µm slices. Afterward, these sections underwent dewaxing hydration, hematoxylin, and eosin (H&E) staining, and sealing. In addition, safranin O fast green staining was performed on the samples, and the Osteoarthritis Research Society International (OARSI) grades were calculated to assess joint destruction. The tissue slides were also stained with TRAP to obverse osteoclast activity; the prescribed steps were as above described (Refer to cell trap staining). For immunohistochemical staining, paraffin sections underwent routine dewaxing, hydration, and thermal antigen retrieval, followed by antigen blocking, incubation with primary and secondary antibodies, then DAB staining, hematoxylin counterstaining, and mounting to observe the expression and localization of autophagy in knee joint tissues. All sections were captured using an Axiovert 40C optical microscope (Zeiss, Germany) and images were analyzed using Image J software. Images and fluorescent intensity profiles were analyzed using ZEN software (ZEISS) and integrated fluorescent intensity was computed using image J software with Coloc2 Plugin.

### RNA Sequencing (RNA‐Seq)

The Trizol kit (Invitrogen, Carlsbad, CA, USA) was used to extract total RNA from RANKL‐induced BMMs with PP2Ac knockdown, according to the manufacturer's protocol.^[^ ^41^ ^]^ The resulting mRNA was fragmented and reverse‐transcribed into cDNA with the NEBNext Ultra RNA Library Prep Kit for Illumina (NEB #7530). After end‐repair, base addition, and ligation with Illumina sequencing adapters, the cDNA fragments were size‐selected by agarose gel electrophoresis and amplified via PCR. The cDNA library was then sequenced on an Illumina Novaseq6000 by Gene Denovo Biotechnology Co. (Guangzhou, China).

### RNA Interference and Transfection

PP2Ac‐specific siRNAs were purchased from Shanghai GenePharma Co., Ltd. BMMs (1.5 × 10^6^ cells per well), and RAW264.7 cells (1 × 10^6^ cells per well) were plated in six‐well plates and incubated overnight. According to the manufacturer's instructions, cells followed by transfection with PP2Ac siRNA or negative control (NC) siRNA. Briefly, dilute 5 µL of RNAi‐Mate reagent with 250 µL of serum‐free DMEM, and leave for 5 min at room temperature. Then 5ul of each siRNA was added and stood for 20 min to form a siRNA/RNAi‐Mate complex. Incubate for 4–6 h and replace with complete medium. All functional experiments with siRNA‐transfected cells were performed after 48 h of transfection. For transfection efficiency experiments, cells were first transiently transfected by NC siRNA or PP2Ac siRNA for 48 h, and then transfected with a green fluorescent protein (GFP) vector and continuously cultured in complete DMEM for an additional 24 h to obverse under a fluorescence microscope. BMMs and RAW264.7 cells were then subjected to subsequent experiments. The specific sequences were included in Table S2 (Supporting Information).

### Gait Analysis

Gait abnormalities in rats were assessed using footprint analysis. Briefly, nontoxic red and blue ink was applied to both hind feet of the rats, and rats were allowed to walk along a custom‐made tunnel on white paper. The left hind limbs were painted with blue ink as the control group, while the right hind limbs were painted with red ink as the OA group. The rats were placed in a quiet environment within a narrow corridor that was 1m long and 10 cm wide and walked three times. The distances and areas of footprints were measured to estimate limb behavioral and functional changes in the knee joint.

### Statistical Analysis

All statistical data were collected by GraphPad Prism 8 software (GraphPad Software Inc., La Jolla, CA) and the outcomes were shown as means ± standard deviations. Both independent samples are normally distributed, Student's *t*‐tests were selected to analyze, otherwise, the Wilcoxon rank test was selected to use. Data from multiple groups satisfied the homogeneity of variance, and one‐way ANOVA analysis was evaluated, if not, the Kruskal Wallis test was selected to compare. A P value of less than 0.05 was considered statistically significant.

## Conflict of Interest

The authors declare no conflict of interest.

## Author Contributions

H.Z., G.G., W.Z., and H.S. contributed equally to this work and are the first co‐authors. All authors were involved in drafting the article or revising it critically for important intellectual content, and all authors approved the final version to be published. H.Z. and D.G. had full access to all of the data in the study and took responsibility for the integrity of the data and the accuracy of the data analysis. X.Y., Y.X., and D.G performed the study conception and designed the study. H.Z., G.G., W.Z., and H.S. acquired data. X.L., Y.X., J.D., Z.W., J.B., X.Y., J.Z. performed the interpretation and analysis of data.

## Supporting information

Supporting Information

## Data Availability

The data that support the findings of this study are available from the corresponding author upon reasonable request.
