# Supplementary material for: PP2Ac Regulates Autophagy via Mediating mTORC1 and ULK1 During Osteoclastogenesis in the Subchondral Bone of Osteoarthritis
Source: Adv Sci (Weinh). 2024 Jul 23;11(36):2404080. doi: 10.1002/advs.202404080 (PMC11423161; doi:10.1002/advs.202404080)
Supplement: Supplementary file 1 — Supporting Information [file ADVS-11-2404080-s001.docx]

**Supplemental Figures and Tables**

**PP2Ac regulates autophagy via mediating mTORC1 and ULK1 during osteoclastogenesis in the subchondral bone of osteoarthritis**

Haifeng Zhang ^1, 2#^, Gaoran Ge ^1#^, Wei Zhang ^1#^, Houyi Sun ^3#^, Xiaolong Liang ^1^, Yu Xia ^1^, Jiacheng Du ^4^, Zerui Wu ^1, 5^, Jiaxiang Bai ^1,6^, Huilin Yang ^1^, Xing Yang ^7*^, Jun Zhou ^1*^, Yaozeng Xu ^1*^, Dechun Geng ^1*^

**Affiliations:**

1 Department of Orthopedics Surgery, the First Affiliated Hospital of Soochow University, Suzhou city, China.

2 Department of Orthopaedic Surgery, Shanghai General Hospital, Shanghai Jiao Tong University School of Medicine, Shanghai, China.

3 Department of Orthopedics, Qilu Hospital of Shandong University, Jinan, China

4 Department of Biochemistry and Molecular Biology, Jeonbuk National University Medical School, Jeonju, Jeonbuk 54896, Korea.

5 Department of Orthopedics, Changshu Hospital Affiliated to Soochow University, Changshu city, China

6 Department of Orthopedics, the First Affiliated Hospital of USTC, Division of Life Sciences and Medicine, University of Science and Technology of China, Hefei city, China

7 Orthopedics and Sports Medicine Center, Suzhou Municipal Hospital, Nanjing Medical University Affiliated Suzhou Hospital, 242, Guangji Road, Suzhou, Jiangsu, China.

# These authors contributed equally to this work.

* **Corresponding Author：**

Professor Dechun Geng, Department of Orthopedics, the First Affiliated Hospital of Soochow University, Suzhou City, China, Email: szgengdc@suda.edu.cn

Professor Yaozeng Xu, Department of Orthopedics, the First Affiliated Hospital of Soochow University, Suzhou City, China, Email: xuyaozeng@163.com

Professor Jun Zhou, Department of Orthopedics, the First Affiliated Hospital of Soochow University, Suzhou City, China, Email: zhou.jun.roy@hotmail.com

Professor Xing Yang, Orthopedics and Sports Medicine Center, Suzhou Municipal Hospital, Suzhou City, China, Email: xingyangsz@126.com

# Haifeng Zhang, Gaoran Ge, Wei Zhang and Houyi Sun contributed contributed equally to this work as co-first authors.

Dechun Geng https://orcid.org/0000-0003-4375-2803

**
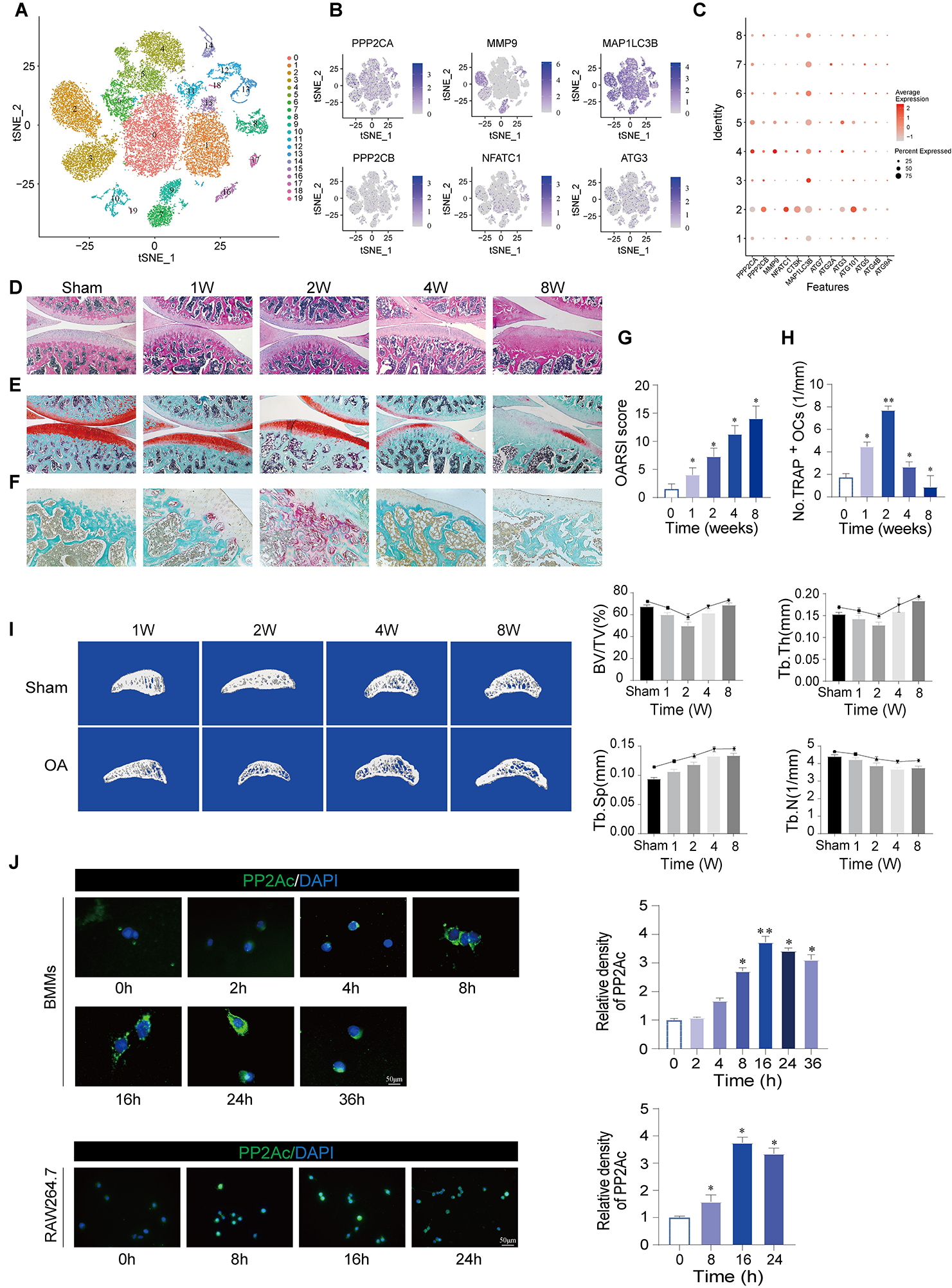
**

**Supplementary Figure S1: A** The t-SNE plot identified 19 clusters from the subchondral bone tissue in OA disease. **B** Feature plots for marker genes of osteoclastic (NFATC1 and MMP9) and autophagy key genes (MAP1LC3B and ATG3) and key genes of PP2Ac (PPP2CA and PPP2CB). The blue color legend shows the normalized expression levels of the genes. **C** Dot plots showing the 13 signature gene expressions across the 8 cellular types (1. B cell; 2. Chondrocytes; 3. Endothelial cells; 4. Macrophage; 5. Monocyte; 6. NK cell; 7. T cell; 8. Tissue stem cells). The size of the dot indicates the proportion of cells expressing particular genes, and the spectrum of colors indicates the average expression level of the marker genes. **D** H&E staining showing morphological change within different time points after ACLT surgery. **E and G** Safranin O fast green staining and OARSI score analysis at various time points in animal OA model. **F and H** The tartrate-resistant acid phosphatase (TRAP) staining and quantitative analysis of positive osteoclast numbers at different time points in the mouse model. **I** The micro-CT 3D observation of subchondral bone trabecular changes at different time points following ACLT surgery and quantitative analysis of bone volume fraction (BV/TV), trabecular number (Tb.N), trabecular thickness (Tb.Th), and trabecular separation (Tb.Sp). **J** Immunofluorescence staining and fluorescence intensity analysis to examine the expression of PP2Ac at different time points in BMMs and RAW264.7 cells during osteoclastogenesis induced by M-CSF and RANKL. (n = 3, mean ± SD; *p < 0.05; **p < 0.01; versus control group).


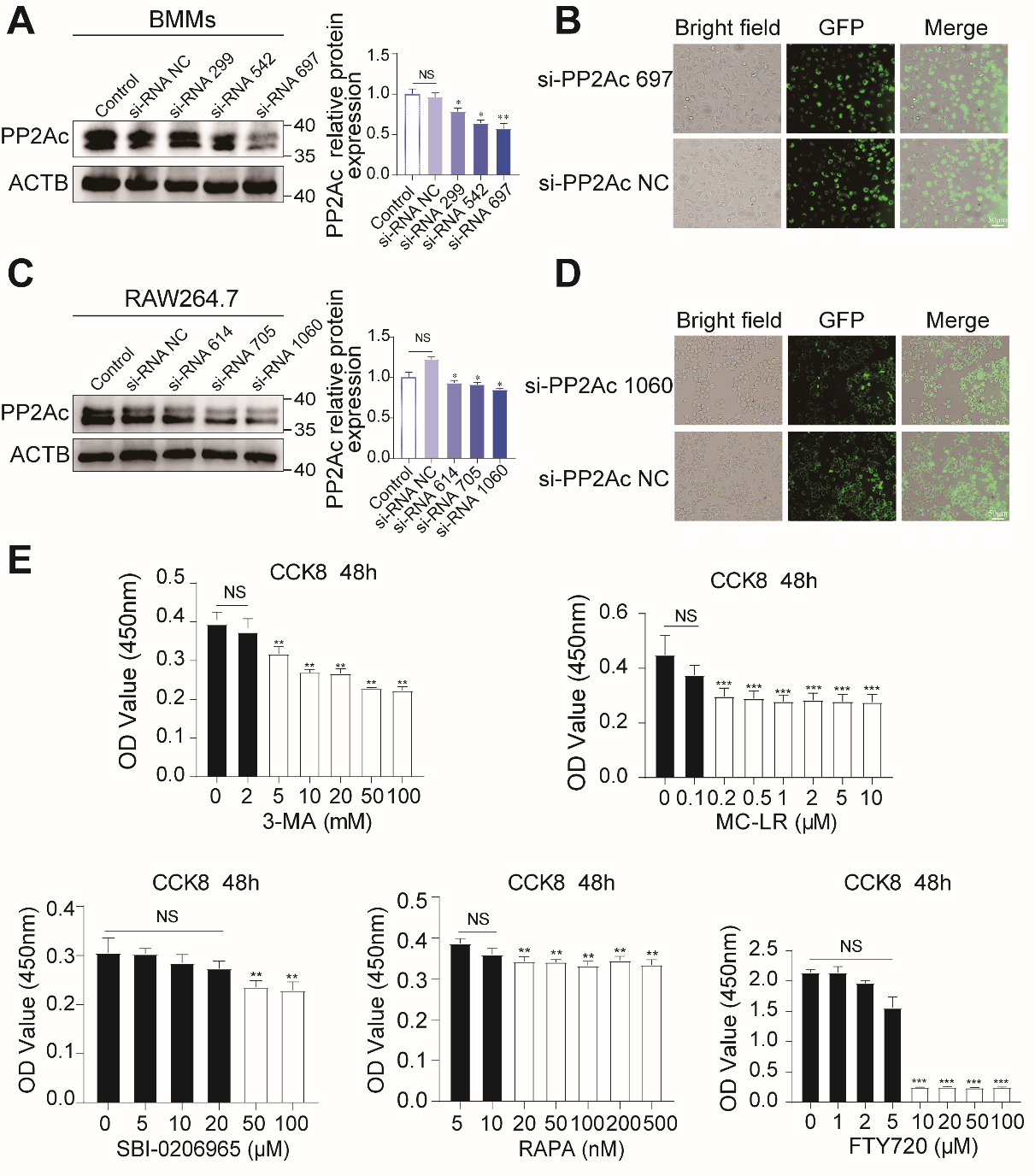


**Supplementary Figure S2: A** Western-blot detection and quantitative analysis to determine the efficacy of PP2Ac knockdown in BMMs. **B** GFP fluorescence observation in BMMs to verify the transfection efficiency of PP2Ac and the cell growth status. **C** Western-blot and quantitative analysis showing the efficiency of PP2Ac knockdown in RAW264.7 cell line. **D** GFP fluorescence detection in RAW264.7 cells to confirm the transfection efficiency of PP2Ac. **E** Cell Counting Kit-8 (CCK 8) assessed cell viability with different reagents in BMMs at 48h. (n = 3, mean ± SD; *p < 0.05; **p < 0.01; ***p < 0.005 versus control group).

**
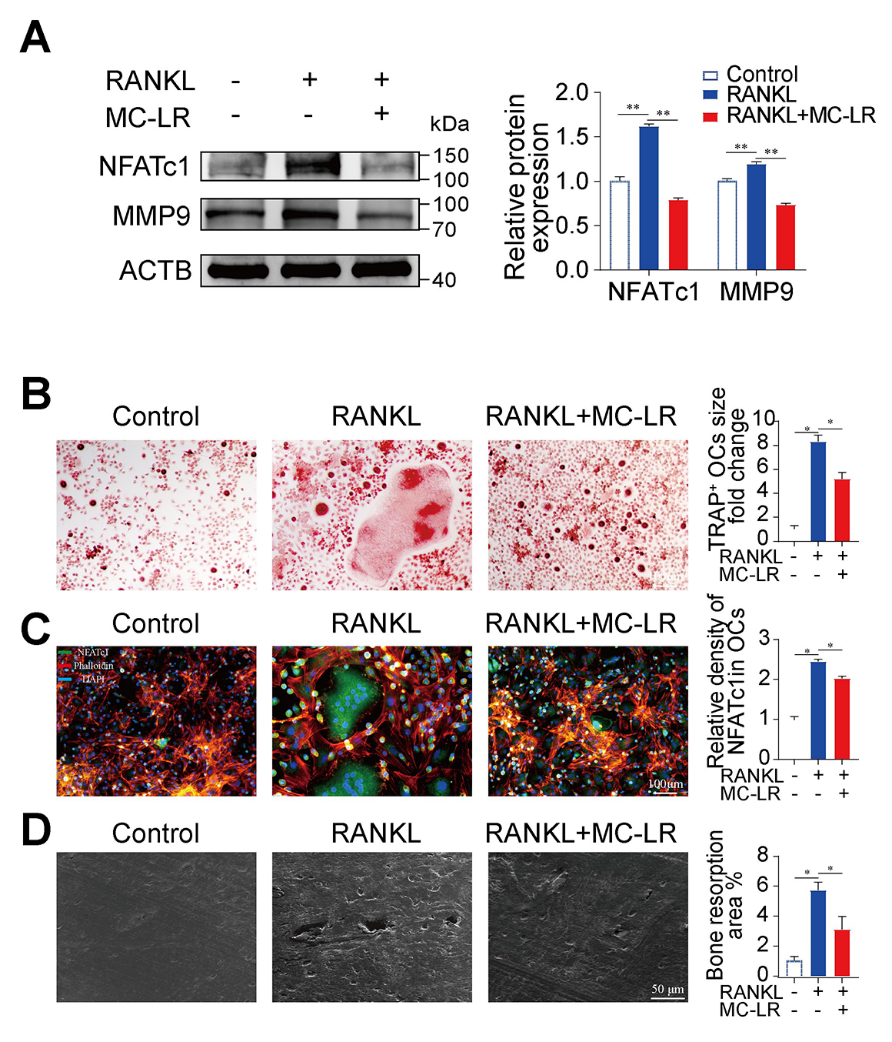
**

**Supplementary Figure S3: A** Western blot assay and gray value quantitative analysis of osteoclast marker expression in BMMs after PP2Ac inhibition. **B** TRAP staining and quantitative analysis TRAP staining to evaluate the size of osteoclasts in RANKL-induced BMMs with PP2Ac inhibition. **C** Immunofluorescence staining and quantitative analysis of NFATc1 with phalloidin or F-actin in RANKL-induced BMMs with PP2Ac inhibition. **D** Bone plate resorption assay and quantitative analysis to quantify the resorption area in BMMs following RANKL induction and inhibition of PP2Ac. (n = 3, mean ± SD; *p < 0.05; **p < 0.01; versus control group).

**
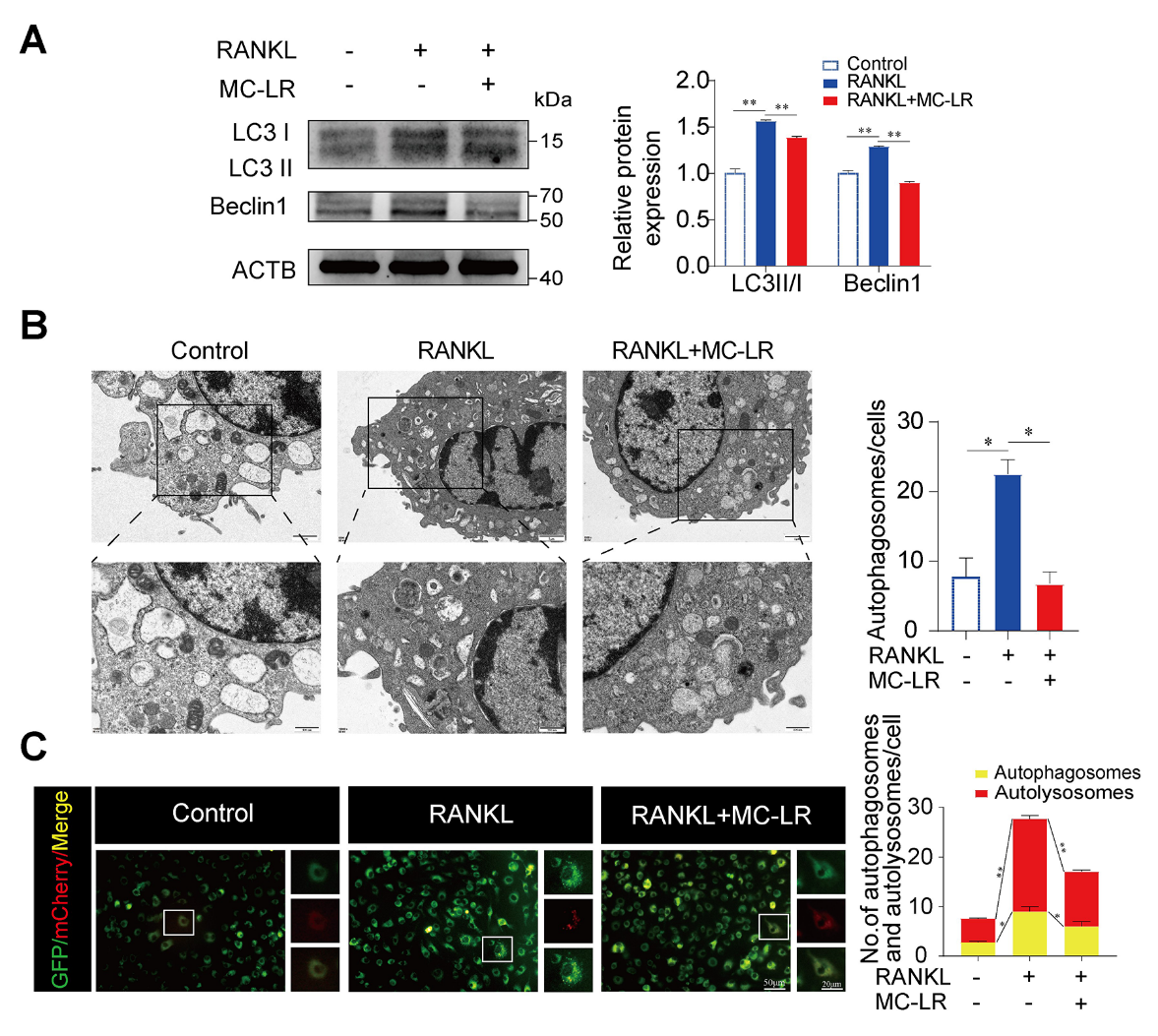
**

**Supplementary Figure S4: A** Western blot experiment and gray value analysis to analyze the expression levels of PP2Ac and autophagy markers in BMMs after RANKL induction. **B** Transmission electron microscopy (TEM) observation and quantitative analysis of autophagosomes and autolysosomes after PP2Ac inhibition in BMMs induced with RANKL. **C** After inhibition of PP2Ac in BMMs, mCherry-GFP-LC3 staining observation and quantitative analysis number of autophagosomes and autophagolysosomes. (n = 3, mean ± SD; *p < 0.05; **p < 0.01; versus control group).

**
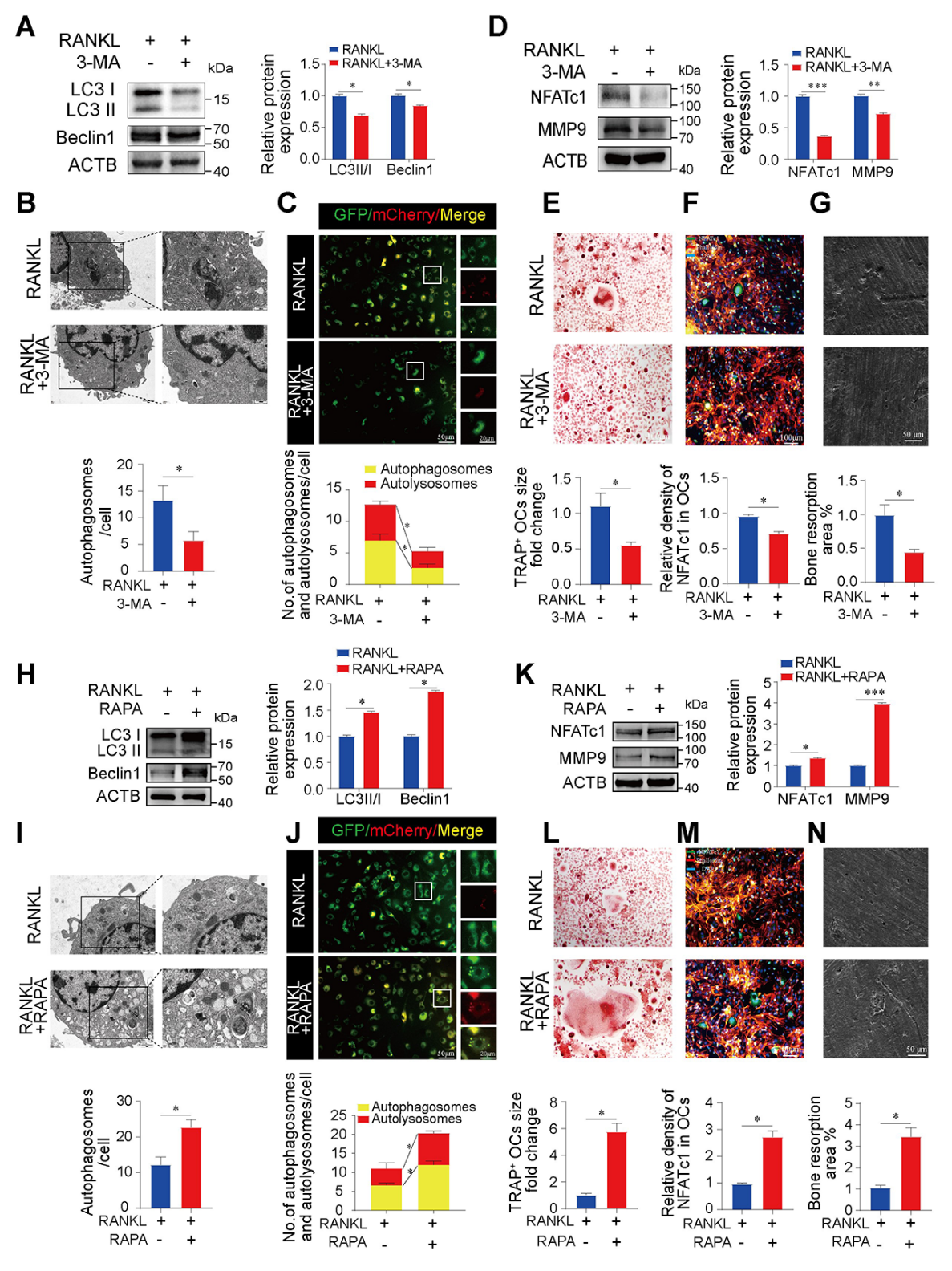
**

**Supplementary Figure S5: A** Autophagy marker protein levels were assessed using western blot assay and gray value analysis following treatment with 3-MA, an autophagy inhibitor, during the process of osteoclastogenesis. **B** The autophagic vacuoles or autophagolysosomes were detected with TEM observation after 3-MA inhibition. **C** The mCherry-GFP-LC3 assay staining and quantification puncta analysis in BMMs after RANKL induction and addition of 3-MA. **D** The protein expression of osteoclast markers was analyzed using western blot after 3-MA intervention in RANKL-induced BMMs. **E** TRAP staining to quantify the size of osteoclasts in RANKL-induced BMMs, both with and without 3-MA intervention. **F** Immunofluorescence staining of NFATc1 and phalloidin associated with analysis of NFATc1 fluorescence intensity between the two groups. G Bone plate resorption assay and quantitative analysis of bone resorption area to identify osteoclast function without or with 3-MA intervation. **H** Western blot analysis and quantitative assessment of gray values to determine the levels of autophagy marker proteins modulated by rapamycin. **I** TEM to detect autophagosomes or autolysosomes in BMMs induced with RANKL or RAPA intervenion. **J** The mCherry-GFP-LC3 staining to detect autophagy dots in BMMs after RAPA intervention. **K** Western blot analysis and quantitative measurement of gray values to investigate the expression of osteoclast marker proteins in BMMs induced with RANKL and treated with RAPA during osteoclastogenesis. **L** TRAP staining and quantitative analysis of positive osteoclast size with or without RAPA intervention. **M** Immunofluorescence staining was conducted for NFATc1 and phalloidin, and fluorescence intensity analysis to compare the NFATc1 levels between two groups. **N** Bone pit resorption assay and assessment of the resorbed area to determine the osteoclast function after inducing BMMs to differentiate into osteoclasts. (n = 3, mean ± SD; *p < 0.05; **p < 0.01; ***p < 0.005 versus control group).


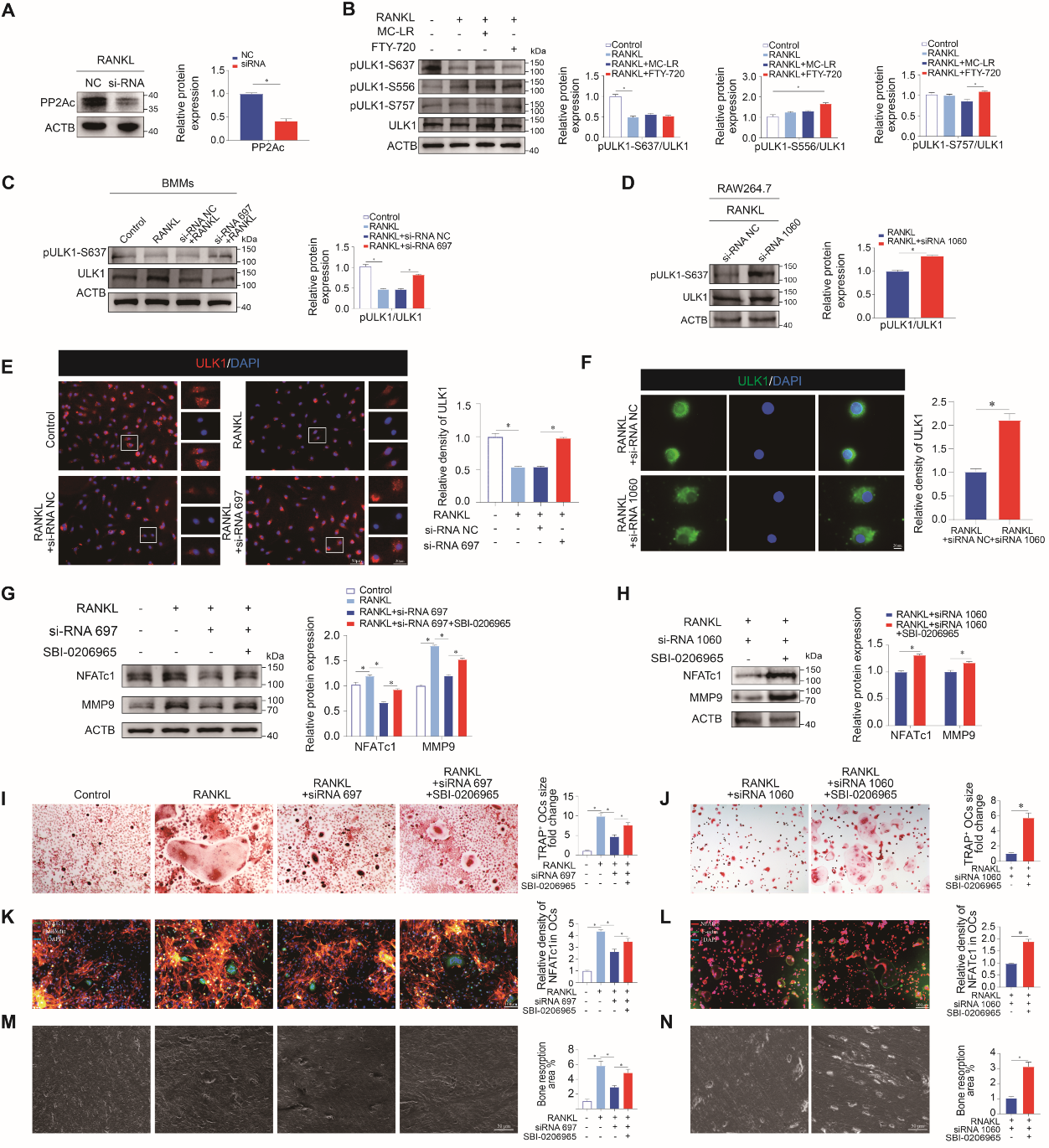


**Supplementary Figure S6: A** Western blot assay suggested that the decrease in PP2Ac expression after knockdown of PP2Ac with RANKL intervention in BMMs. **B** Western blot experiment showing the protein expression levels of the S637, S556 and S758 phosphorylation sites of ULK1 in BMMs treated with PP2Ac inhibitors and activators. **C-D** Western bolt and quantitative analysis revealed the level of phosphorylated ULK1 at S637 in BMMs and RAW264.7 cells with PP2Ac knockdown. **E-F** Immunofluorescence staining and quantitative analysis of ULK1 fluorescence intensity to evaluate the effect of PP2Ac knockdown on BMMs and RAW264.7 cell line. **G-H** Western blot and gray value analysis of osteoclast marker protein expression with ULK1 inhibition based on PP2Ac knockdown in BMMs and RAW264.7 cell line. **I-J** TRAP staining and positive osteoclasts counting with ULK1 inhibition based on PP2Ac knockdown in BMMs and RAW264.7 cell line. **K-L** NFATc1 with phalloidin or F-actin immunofluorescence staining and fluorescence intensity analysis with ULK1 inhibition under PP2Ac knockdown in BMMs and RAW264.7 cell line. **M-N** Bone plate resorption assay and quantitative analysis of resorption area with ULK1 inhibition after PP2Ac knockdown in BMMs and RAW264.7 cell line. (n = 3, mean ± SD; *p < 0.05; **p < 0.01; versus control group).


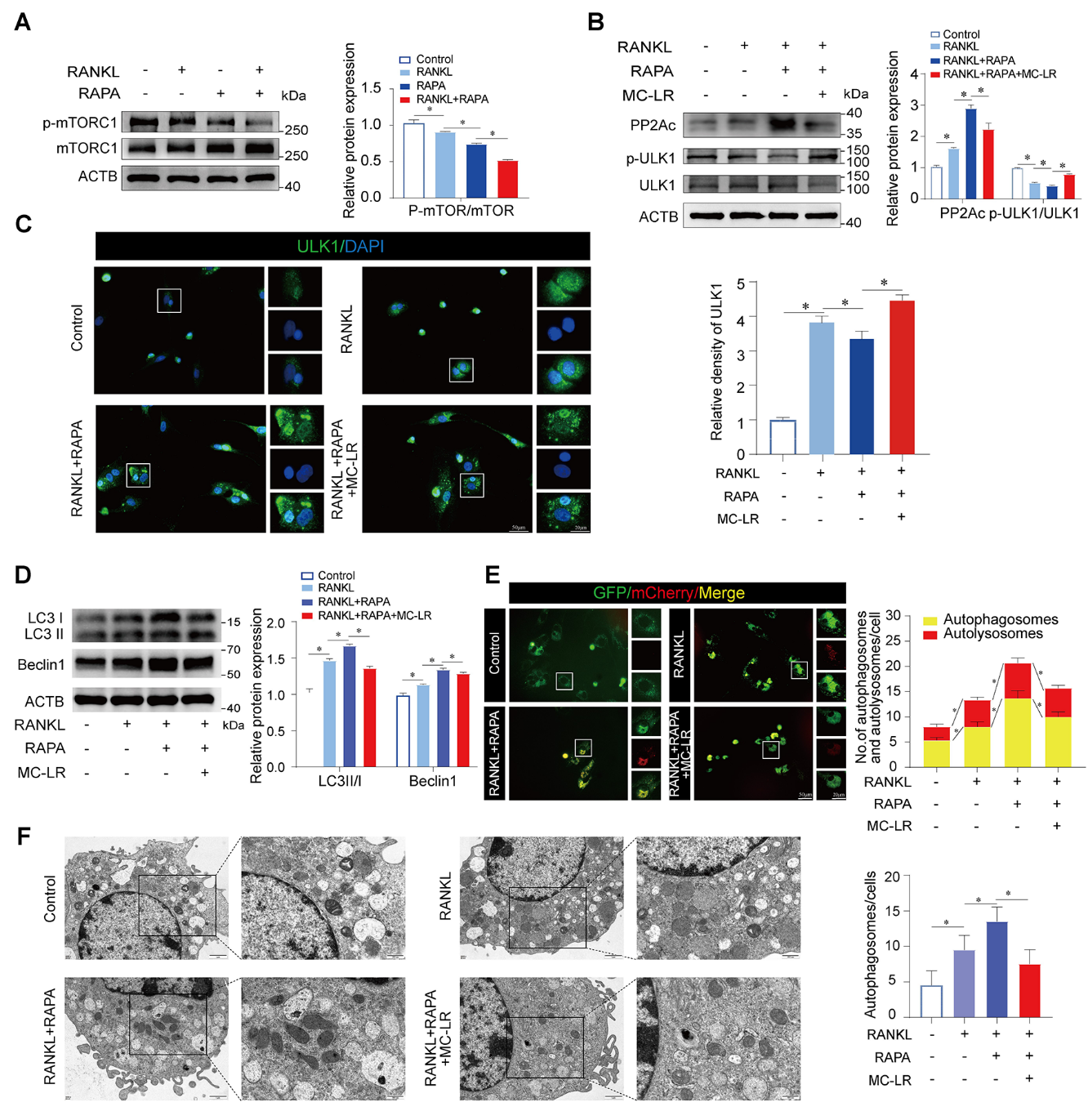


**Supplementary Figure S7: A** The expression level of mTORC1 phosphorylation was analyzed using western blot and gray value analysis with RANKL induction and mTORC1 inhibitor during osteoclastogenesis. **B** Western blot and quantitative analysis of PP2Ac and ULK1 protein expression under mTORC1 inhibition with MC-LR on RANKL induced BMMs. **C** Immunofluorescence staining of ULK1 based on mTORC1 inhibition and PP2Ac activity inhibition in RANKL-induced BMMs. **D** Western blot experiments and gray value analysis to detect changes in the expression of autophagy markers upon inhibiting PP2Ac activity with the addition of RAPA. **E** The mCherry-GFP-LC3 assay and fluorescence quantitative analysis to detect autophagosomes or autolysosomes in RANKL-induced BMMs with RAPA or MC-LR intervention. **F** TEM observation and quantitative analysis of autophagosome or autophagolysosome alteration in RANKL-induced BMMs under mTORC1 and PP2Ac inhibition. (n = 3, mean ± SD; *p < 0.05; **p < 0.01).


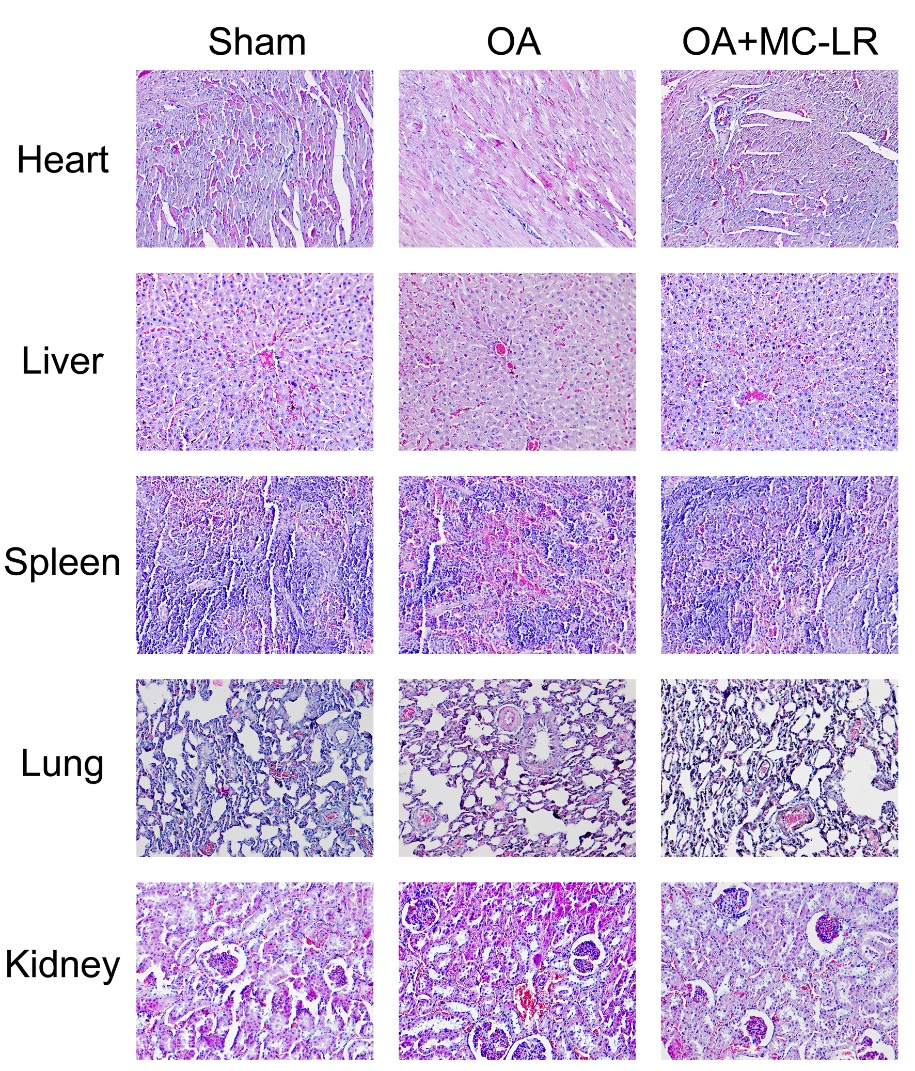


**Supplementary Figure S8:** H&E staining was performed to observe the toxicity of MC-LR to the heart, liver, spleen, lungs, and kidneys in rats. No apparent toxicity events were observed in the rats treated with the MC-LR.

**
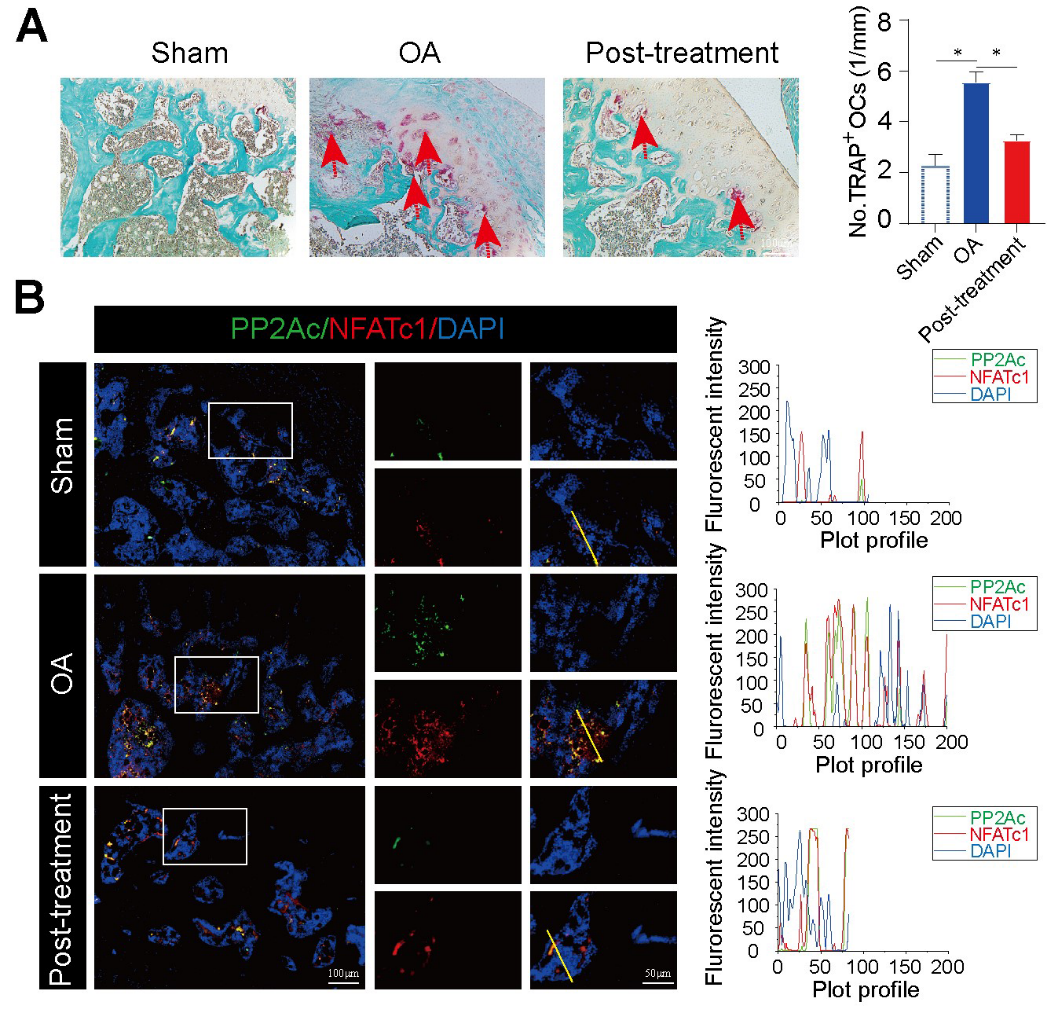
**

**Supplementary Figure S9: A** TRAP staining and quantitative analysis to assess the number of positive osteoclasts in the subchondral bone among different groups. **B** Immunofluorescence co-localization of PP2Ac and NFATc1, as well as fluorescence intensity analysis, to evaluate in the rat OA model group and the treatment group with PP2Ac inhibition. (n = 5, mean ± SD; *p < 0.05; **p < 0.01; versus sham group).

**
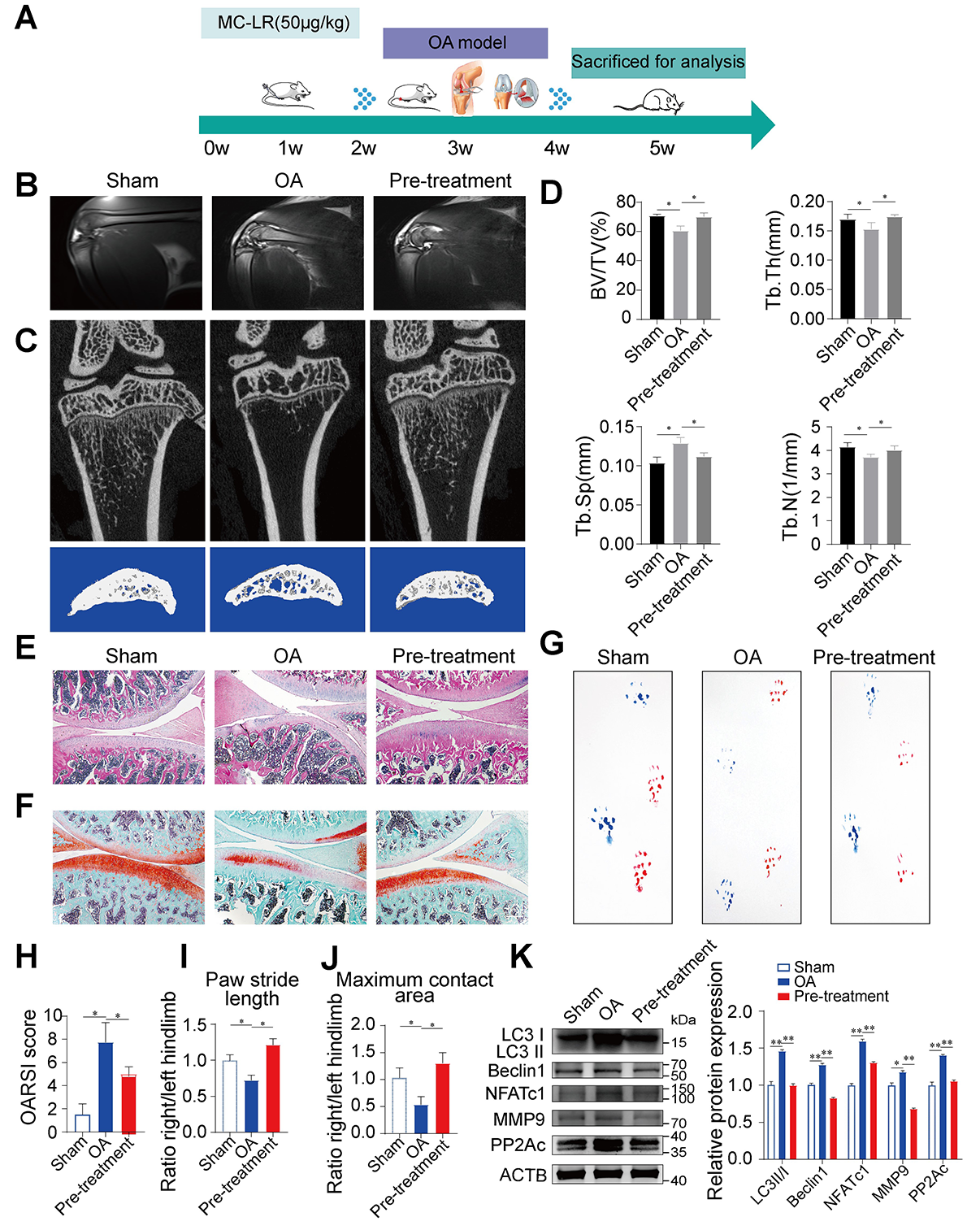
**

**Supplementary Figure S10: A** The flow chart depicted the preventive interventions in the rat OA model. **B** The MRI T2-weighted image to illustrate the signal changes in the knee joint cartilage and subchondral bone of the rat OA model. **C** Micro-CT scans and 3D reconstructions (in coronal and sagittal views) to visualize the morphology of subchondral trabecular bone. **D** Quantitative assessment of trabecular parameters, including BV/TV, Tb. N, Tb.Th, and Tb. Sp in different groups. **E** H&E staining showing the histological changes after pre-treatment with the PP2Ac inhibitor. **F** Comparison of histomorphology between the articular cartilage and subchondral trabecular bone using Safranin O fast green staining. **G** The inked footprints gait map assessing the functional recovery of the rats knee joint. **H** The OARSI score showed variation among groups with pre-treatment model. **I-J** The hind limb gait parameters, such as stride length and contact area, to assess changes and performance in gait. **K** Western blots showing the alterations in the expression levels of osteoclast markers, autophagy markers, and key molecules of PP2Ac. (n = 5, mean ± SD; *p < 0.05; **p < 0.01; versus sham group).

**
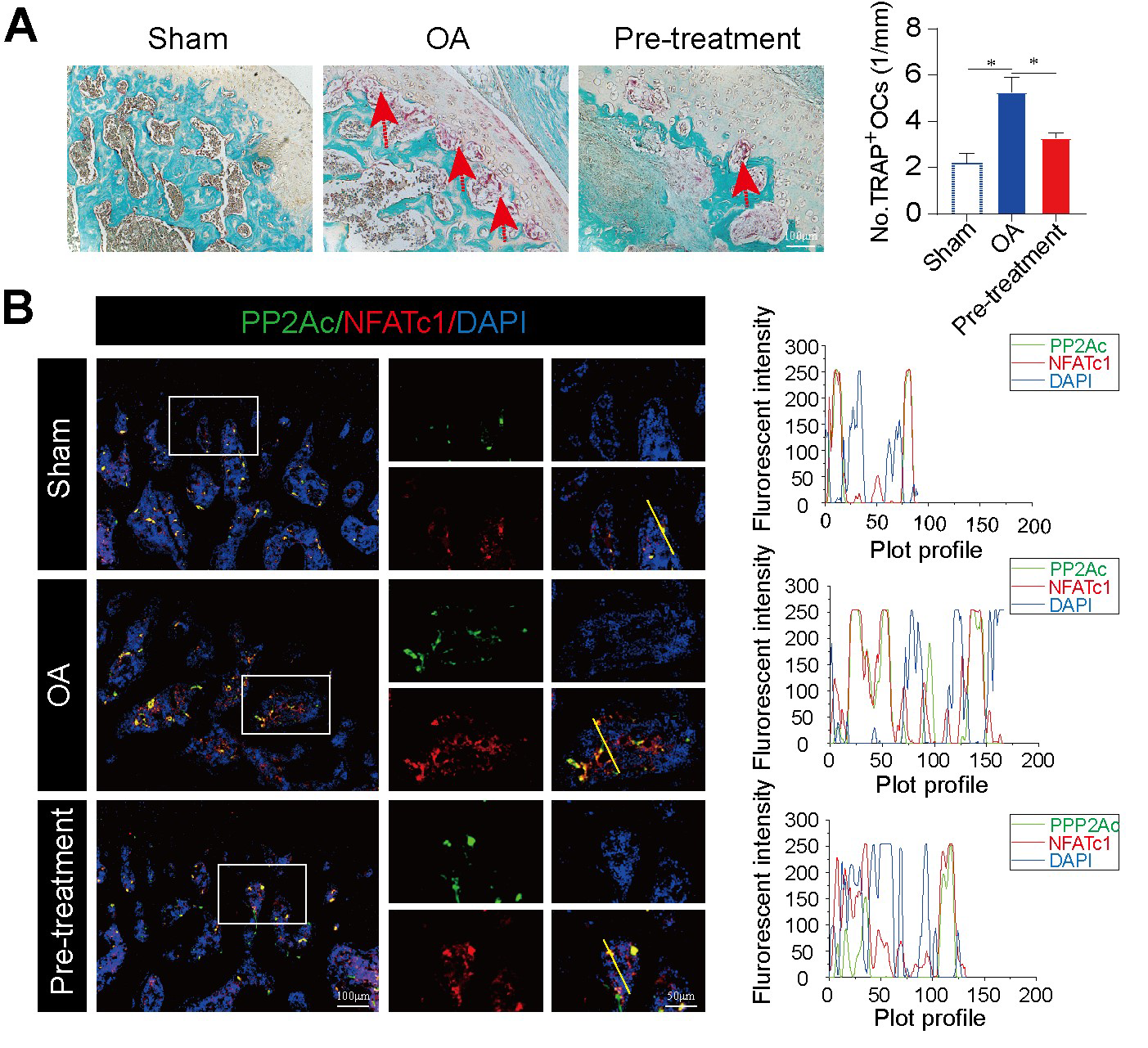
**

**Supplementary Figure S11: A** TRAP staining and quantitative analysis of positive osteoclasts in the subchondral bone to estimate among different groups in the pre-prevention model. **B** PP2Ac and NFATc1 immunofluorescence co-localization and fluorescence intensity analysis in rat OA model group and pre-treatment group with PP2Ac inhibition. (n = 5, mean ± SD; *p < 0.05; **p < 0.01; versus sham group).

**Supplementary table S1.** Clinical information

**Supplementary Table S2.** RNA sequences
